# RNA expression of 6 genes from metastatic mucosal gastric cancer serves as the global prognostic marker for gastric cancer with functional validation

**DOI:** 10.1038/s41416-024-02642-6

**Published:** 2024-03-11

**Authors:** Yun-Suhk Suh, Jieun Lee, Joshy George, Donghyeok Seol, Kyoungyun Jeong, Seung-Young Oh, Chanmi Bang, Yukyung Jun, Seong-Ho Kong, Hyuk-Joon Lee, Jong-Il Kim, Woo Ho Kim, Han-Kwang Yang, Charles Lee

**Affiliations:** 1 Department of Surgery, Seoul National University College of Medicine, Seoul, South Korea; 2 Department of Surgery, Seoul National University Hospital, Seoul, South Korea; 3 Department of Surgery, Seoul National University Bundang Hospital, Seongnam, South Korea; 4 The Jackson Laboratory for Genomic Medicine, Farmington, CT 06032 USA; 5 Cancer Research Institute, Seoul National University College of Medicine, Seoul, South Korea; 6 Center for Supercomputing Applications, Division of National Supercomputing, Korea Institute of Science and Technology Information, Daejeon, South Korea; 7 Department of Biomedical Sciences, Seoul National University College of Medicine, Seoul, South Korea; 8 Genomic Medicine Institute, Medical Research Center, Seoul National University College of Medicine, Seoul, South Korea; 9 Department of Pathology, Seoul National University College of Medicine, Seoul, South Korea

**Keywords:** Cancer genomics, Predictive medicine, Gastric cancer

## Abstract

**Background:**

Molecular analysis of advanced tumors can increase tumor heterogeneity and selection bias. We developed a robust prognostic signature for gastric cancer by comparing RNA expression between very rare early gastric cancers invading only mucosal layer (mEGCs) with lymph node metastasis (Npos) and those without metastasis (Nneg).

**Methods:**

Out of 1003 mEGCs, all Npos were matched to Nneg using propensity scores. Machine learning approach comparing Npos and Nneg was used to develop prognostic signature. The function and robustness of prognostic signature was validated using cell lines and external datasets.

**Results:**

Extensive machine learning with cross-validation identified the prognostic classifier consisting of four overexpressed genes (HDAC5, NPM1, DTX3, and PPP3R1) and two downregulated genes (MED12 and TP53), and enabled us to develop the risk score predicting poor prognosis. Cell lines engineered to high-risk score showed increased invasion, migration, and resistance to 5-FU and Oxaliplatin but maintained sensitivity to an HDAC inhibitor. Mouse models after tail vein injection of cell lines with high-risk score revealed increased metastasis. In three external cohorts, our risk score was identified as the independent prognostic factor for overall and recurrence-free survival.

**Conclusion:**

The risk score from the 6-gene classifier can successfully predict the prognosis of gastric cancer.

## Introduction

Gastric cancer is the fifth most common malignancy and the third leading cause of cancer-related death in the world [1]. The high risk of invasion and metastasis including regional lymph node metastasis, is the most important prognostic feature to explain such aggressiveness. Previous studies to identify additional prognostic markers for gastric cancer usually have focused on large, advanced tumors because of the power to detect overexpressed genes and the availability of tumor tissue [2–5]. The gene expression profiles of these samples were then compared to tumors of different phenotypes without proper matching of baseline characteristics [4, 6, 7]. As tumors progress to more advanced stage, confounding factors including different clinicopathologic features not related to essential tumor biology or increased tumor heterogeneity are also likely to be accumulated, which eventually reduce the robustness of the derived molecular signatures [8, 9]. Besides, even though baseline characteristics were matched, the studies often had a limited number of samples or samples only from the certain high stage [10].

On the other hand, the comparison of early-stage cancer with significantly different prognostic features may solve this selection bias. However, it is difficult to obtain enough volume of small, early-stage tumors with or without significant invasion and metastatic features. Also procurement of those tumors would be very limited after meticulous pathological processes at the clinic. Lymph node (LN) metastasis is one of the most critical poor prognostic features of gastric cancer, even in early gastric cancer [11, 12]. Early gastric cancers confined to the mucosa (mEGC) could be the earliest stage which still can show LN metastasis [13, 14]. Our previous large-scale clinicopathologic study analyzing 1003 mEGC reported 1.8% of LN metastasis [15]. Propensity score matching of mucosal gastric cancers with LN metastasis to those without LN metastasis could minimize the risk of selection bias and tumor heterogeneity. We posited that a careful application of statistical analyses and machine learning models of gene expression profiles of matched tumor samples would enable us to develop a robust prognostic signature for gastric cancer patients. Along with the functional validation, this study provides evidence for the robustness of the risk score as the independent prognostic marker in three external cohorts.

## Results

### Feature selection for mucosal early gastric cancer with lymph node metastasis

**Fig. 1:**
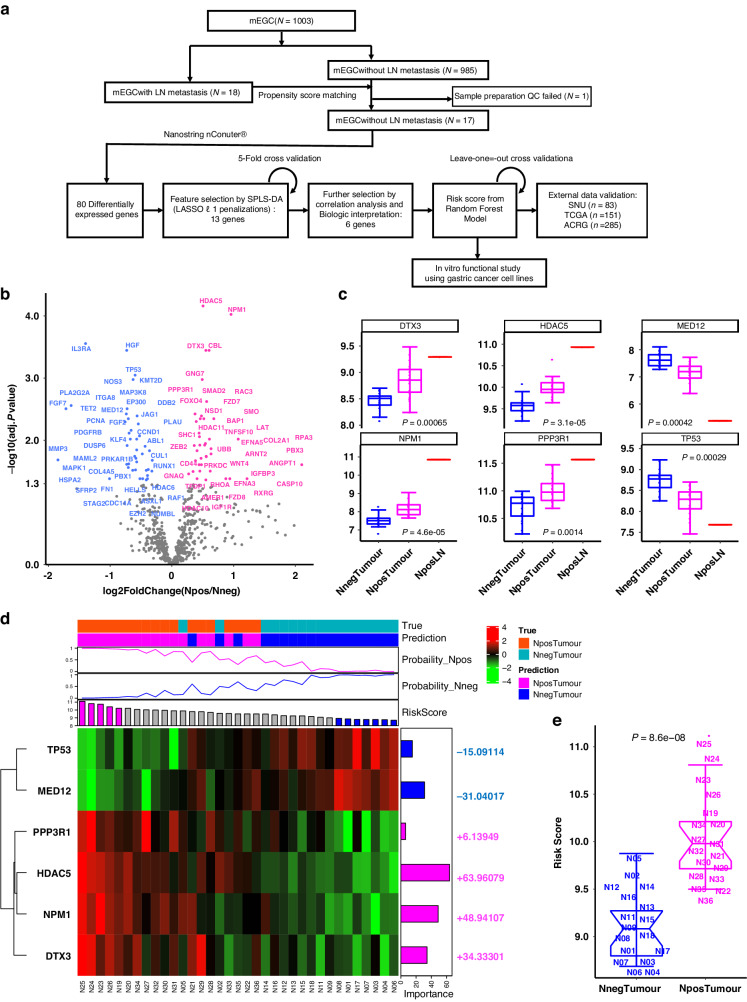
Development of 6-gene classifier predicting gastric cancer confined within mucosal layer with lymph node metastasis (Npos), and those without LN metastasis (Nneg). **a** A diagram of study design. **b** 80 differentially expressed genes with adjusted *P* value < 0.05 between Npos and Nneg. **c** RNA expression of six genes among Npos, Nneg, and metastatic lymph node of Npos (NposLN). **d** A heatmap demonstrating scaled expression of 6-gene classifier with variable importance retrieved from Random Forest model classifying Npos and Nneg. Annotations as prediction and probability represent the information by Random Forest. Magenta and blue bars in the risk score represent samples whose risk score was 1 standard deviation above or below the mean value. **e** Comparison of the risk score between Npos and Nneg.

Out of previously identified 1003 mEGCs that were surgically resected in Seoul National University (SNU) Hospital, we retrieved all mEGCs with lymph node metastasis (Npos) (*n* = 18). We matched mEGCs without LN metastasis (Nneg) using 1:1 propensity score matching with age, sex, tumor size, tumor location, and Lauren classification as covariates (Fig. 1a) [15]. After meticulous laboratory quality control, one Nneg sample was excluded, and RNA expression of of 18 Npos, 17 Nneg, and 1 metastatic lymph node (NposLN) samples were profiled using Nanaostring platform (Supplementary Table S1).

Comparison of Npos and Nneg samples identified 80 differentially expressed genes (DEG) at a false discovery rate of 5% (Fig. 1b). Canonical pathway analysis of the 80 DEGs demonstrated that regulation of the epithelial-mesenchymal transition (EMT) pathway including WNT4 and FZD was the most significantly enriched pathway (*P* = 3.162278e-12) (Supplementary Table S2). Wnt/β-catenin signaling was also significantly enriched along with the co-overexpression of WNT4 and Frizzled receptor signal (*P* = 1.584893e−09).

For feature selection, we used Lasso ${l}_{1}$ penalization with spares Partial Least Squares regression-Discriminant Analysis (sPLS-DA) for 80 DEGs [16]. After 5-fold cross-validation, 2 PLS components, including 13 and 40 genes, were selected based on the lowest balanced error rate (Supplementary Fig. S1). For a more consistent prognostic classifier, we analyzed Spearman correlation for 13 genes including HDAC5, NPM1, IL3RA, HGF, TP53, CBL, DTX3, GNG7, KMT2D, MED12, FGF7, SMAD2, and PPP3R1 in the first PLS component, which eventually led to 8 genes (HDAC5, NPM1, TP53, CBL, DTX3, KMT2D, MED12, and PPP3R1) with *P* < 0.001 (Supplementary Fig. S2). Out of those 8 genes, CBL, is shown to ubiquitinate nuclear β-catenin to switch off the Wnt signaling [17, 18]. Considering the Wnt activation level coupled with main regulation EMT pathway was higher in Npos cells compared with Nneg cells (Supplementary Table S2), it is reasonable that CBL could be upregulated as a negative feedback in the Wnt activated cells like other Wnt suppressor NOTUM [19]. KMT2D, lysine-specific methyltransferase 2D that adds a trimethylation mark to H3K4, has been known to be inhibited by the overexpression of HDACs including HDAC5 [20, 21]. Considering the significantly enriched Wnt/β-catenin signaling pathway and the most confident significance of HDAC5 from DEG analysis, we excluded CBL and KMT2D as a negative feedback or secondary bystander followed by Wnt/β-catenin signaling or overexpression of HDAC5. Finally, six genes including HDAC5, NPM1, DTX3, PPP3R1, TP53, and MED12 were selected as the signature classifiers to predict poor prognosis of gastric cancer. The remaining six genes, HDAC5, NPM1, DTX3, PPP3R1, TP53, and MED12, were used to derive a risk score capable of predicting the prognosis of gastric cancer. Among these genes, HDAC5, NPM1, DTX3, and PPP3R1 showed increasing expression, and TP53 and MED12 showed decreasing expression across Nneg, Npos, and NposLN (Fig. 1c).

### Developing the risk score model using machine learning

We used a Random Forest prediction model to develop a predictor of Npos status based on the six genes as features. We attained 88.89% sensitivity, 94.12% specificity, and 91.5% balanced accuracy based on the leave-one-out cross-validation strategy (Supplementary Table S3). However, this model did not apply to tumors where the gene expression profiles were assayed using a different platform (Supplementary Fig. S3). Therefore, we calculated the tumor progression “risk score” per sample within each cohort as the weighted sum of the expression of six genes, where the weights are the variable importance from the Random Forest model and the directionality of expression in the test dataset (Fig. 1d) (Supplementary Table S4). The risk score distribution was unimodal without serious skewness, irrespective of various RNA expression platforms (Supplementary Fig. S3). As the risk score was above the mean +1 standard deviation (SD) or below the mean – 1 SD, the probability of classifying Npos or Nneg by Random Forest approached certainty (probability of 1 or 0) asymptotically (Fig. 1d). The risk score of Npos was significantly higher than that of Nneg (*P* = 8.6e−8) (Fig. 1e).

### Loss of TP53, MED12 and gain of HDAC5, NPM1, DTX3 and PPP3R1 promote gastric EMT, tumor invasiveness and drug resistance

**Fig. 2:**
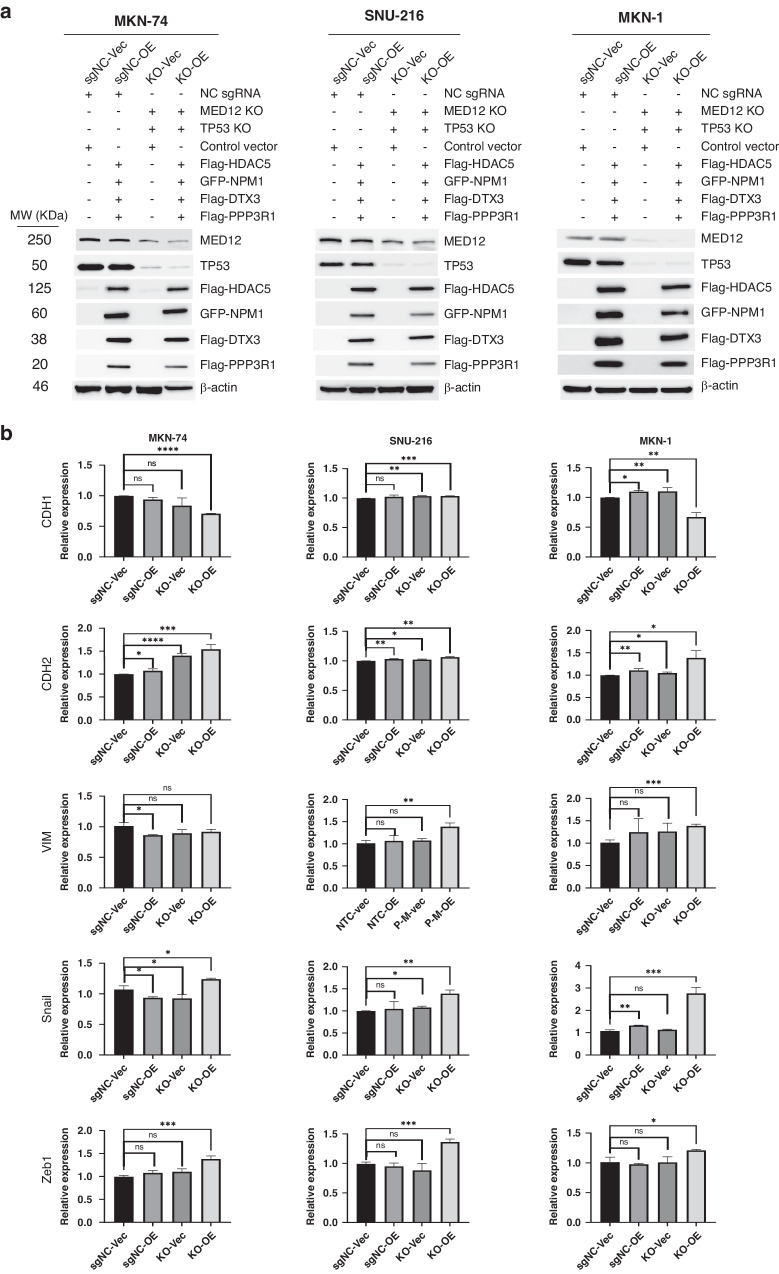
TP53/MED12 double Knockout (KO) and co-overexpression (OE) of HDAC5, NPM1, DTX3 and PPP3R1 promotes gastric EMT. **a** TP53/MED12 double KO were performed using the Lenti-CRISRPR/cas9 system then Flag or GFP tagged PPP3R1, HDAC5, NPM1 and DTX3 were co-transfected in TP53/MED12 double KO MKN-74, SNU-216 and MKN-1 gastric cancer cell. Each protein expression was confirmed by western blot analysis. **b** The mRNA expression of EMT associated gene CDH1, CDH2, VIM, Snail and Zeb1 were detected by q-PCR analysis in sgNC-Vec (the control vector transfection in the negative control sgRNA infected cell), sgNC-OE (HDAC5, NPM1, DTX3 and PPP3R1 co-transfection in the negative control sgRNA infected cell), KO-Vec (the control vector transfection in TP53/MED12 double KO) and KO-OE (HDAC5, NPM1, DTX3 and PPP3R1 co-transfection in TP53/MED12 double KO) cells. *P < 0.05; **P < 0.01; ***P < 0.001; ****P < 0.0001.

**Fig. 3:**
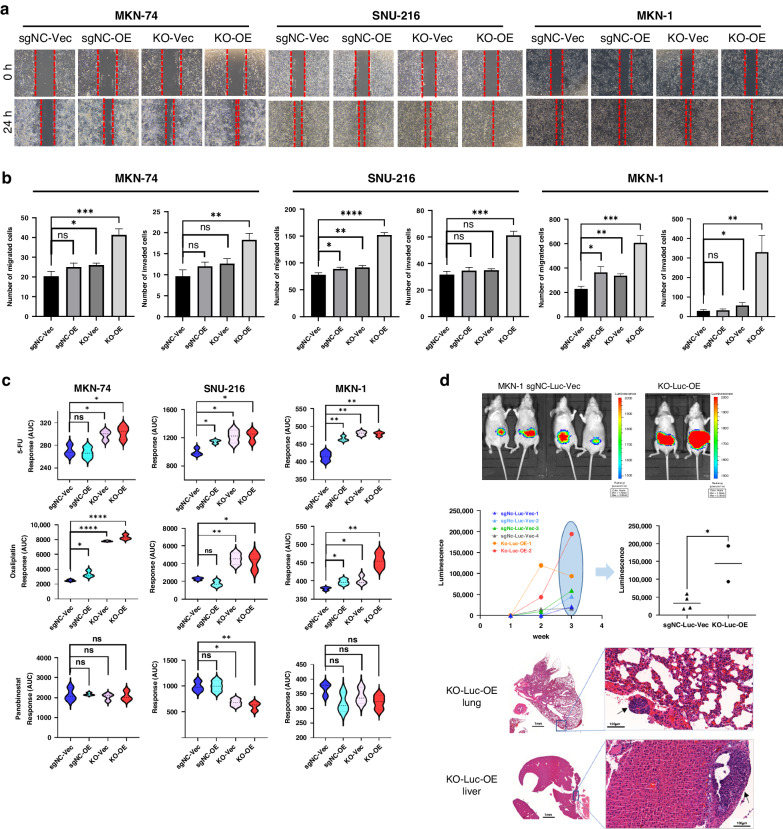
TP53/MED12 double Knockout (KO) and co-overexpression (OE) of HDAC5, NPM1, DTX3 and PPP3R1 promote tumor aggressiveness and drug resistance. **a** The functional changes according to the expression of six target genes were analyzed by wound scratch assay. **b** The effect of TP53/MED12 double KO and/or co-OE of HDAC5, NPM1, DTX3 and PPP3R1 on the migration and invasion of MKN-74, SNU-216 and MKN-1 cells was evaluated by Transwell assay. **c** Effect of chemotherapeutic drugs including 5-Fluorouracil (5-FU), Oxaliplatin and Panobinostat (HDAC inhibitor), was tested in TP53/MED12 double KO and/or HDAC5, NPM1, DTX3 and PPP3R1 co-overexpressed MKN-74, SNU-216 and MKN-1 cells. The drug response was evaluated by area under the fitted dose response curve (AUC). **d** In vivo experiments of the MKN-1 cells (control and KO-OE lines) to nude mice’s tail vein to examine the metastasis. Luminescence imaging of mice at 3 weeks following tail vein injection of MKN-1-sgNC-Luc-Vec (control) or MKN-1-KO-Luc-OE (KO-OE line) (upper panel). Luminescent activity in the region of interest (ROI) was presented as weekly data for 3 weeks and as individual data points at week 3 (middle panel). Hematoxylin and eosin (H&E) staining image of MKN-1-KO-Luc-OE-2 mouse lung and liver. Black arrows indicate metastatic human gastric cancer cells (bottom panel). All in vitro experiment was performed in triplicated, and the mean values are presented. *P < 0.05; **P < 0.01; ***P < 0.001. sgNC-Vec (the control vector transfection in the negative control sgRNA infected cell), sgNC-OE (HDAC5, NPM1, DTX3 and PPP3R1 co-transfection in the negative control sgRNA infected cell), KO-Vec (the control vector transfection in TP53/MED12 double KO) and KO-OE (HDAC5, NPM1, DTX3 and PPP3R1 co-transfection in TP53/MED12 double KO).

We computed the risk scores of 37 gastric cancer cell lines to elucidate the six target gene expressions’ consequences. We selected MKN-74 (low-risk score), SNU-216 (middle-risk score), and MKN-1 (high-risk score) for in vitro experiments (Supplementary Fig. S4). We initially made stable TP53/MED12 double knockout (KO) cells using a lentiviral-based CRISPR/Cas9 system and examined co-overexpression (OE) of HDAC5, NPM1, DTX3, and PPP3R1 in each cell line (Fig. 2a). To identify whether loss of TP53/MED12 and gain of HDAC5, NPM1, DTX3, and PPP3R1 altered gastric EMT, expression of several EMT maker genes was evaluated (Fig. 2b). In TP53/MED12 double KO and four gene co-OE (KO-OE) MKN-74 and MKN-1 cells, CDH1 mRNA expression was significantly decreased whereas CDH2 mRNA expression increased compared with each control cell (sgNC-Vector, sgNC-OE or KO-Vec only). Vimentin expression was significantly increased in KO-OE SNU-216 and MKN-1 cells compared with each control cell. Snail and Zeb1 mRNA expression were significantly increased in all KO-OE cells compared with each control cell. We performed a wound healing assay and migration assay to investigate the effect of KO-OE on cell migration. The distance between wound edges of KO-OE MKN-74, SNU-216, and MKN-1 cells dramatically decreased than those of control cells in 24 h (Fig. 3a). In addition, each KO-OE cell line presented a significantly increased number of migrated cells as well as invasiveness compare with its control cell line (Fig. 3b, Supplementary Fig. S5). Next, we tested drug sensitivity for 5-Fluorouracil (5-FU), Oxaliplatin, and Panobinostat (a pan-histone deacetylase inhibitor) in KO-OE MKN-74, SNU-216, and MKN-1 cells. Regarding 5-FU and Oxaliplatin, a standard cytotoxic chemotherapeutic regimen for gastric cancer, the area under the dose-response curve (AUC) (Supplementary Fig. S6) significantly increased in all KO-OE MKN-74, SNU-216 and MKN-1 cells compared with their control cell (Fig. 3c). However, AUC of Panobinostat was not increased in KO-OE MKN-74 or MKN-1, and even significantly decreased in KO-OE SNU-216. These results suggested that double KO of TP53/MED12 and co-OE of HDAC5, NPM1, DTX3, and PPP3R1 functionally enhanced migration and invasion potential of gastric cancer cells, facilitated EMT and increased the resistance against standard cytotoxic chemotherapeutic drugs for gastric cancer.

Additionally, we confirmed the metastatic potential of the six genes in in vivo experiments. MKN-1 sgNC cells stably expressing luciferase (MKN-1-sgNC-Luc) and MKN-1 TP53/MED12 double KO cells stably expressing luciferase (MKN-1-KO-Luc) were established by luciferase-expressing lentivirus infection. 5 × 10^5^ of each cell that luciferase-expressing-control or KO-OE cells were injected into the tail vein of six-week-old female nude mice (Control group *n* = 4, KO-Luc-OE group *n* = 2). The quantification of luciferase activity was measured once a week after cell injection using an IVIS image analyzer. Luciferase activity was detected in the abdominal cavity of all mice 3 weeks after cell injection (Fig. 3d, upper panel). Two weeks after cell injection, the KO-Luc-OE group exhibited an average luciferase activity that was 14 times higher than that of the control group (*P* = 0.0336). Moreover, mice administered with KO-Luc-OE cells demonstrated a 4.1-fold increase in luciferase activity compared to the control group (*P* = 0.0338) 3 weeks after cell injection (Fig. 3d, middle panel). All mice were sacrificed 3 weeks after cell injection. Metastatic human tumor cells were observed in the lung and liver of KO-Luc-OE mice (Fig. 3d, bottom panel and Supplementary Fig. S7)

### External dataset validation and prognostic implication of risk score

**Fig. 4:**
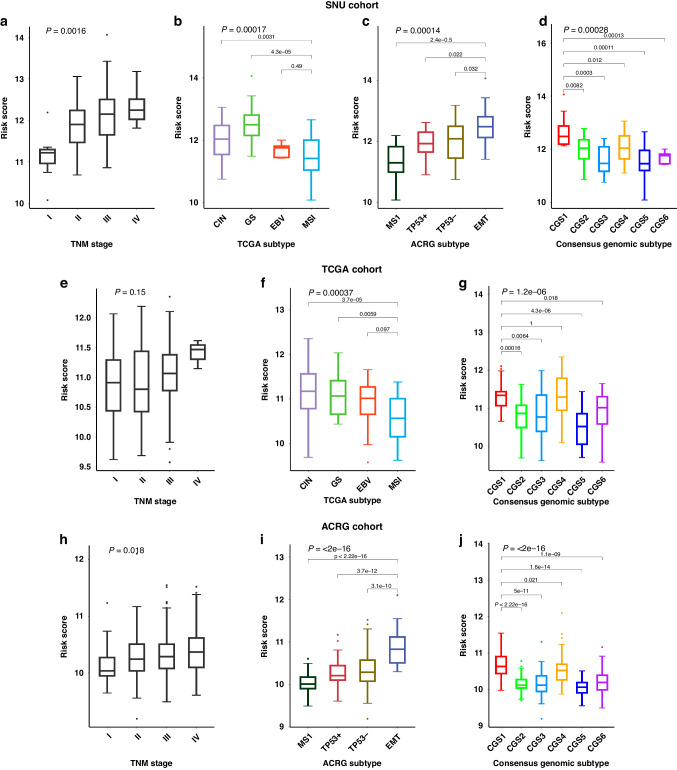
The comparison of risk score with other molecular subtypes of gastric cancer. Boxplot of risk scores (y-axis) between TNM stage and other molecular subtypes were presented. **a**–**d** In the SNU cohort, risk scores were compared within TNM stage and TCGA, ACRG, and CGS subtypes. **e**–**g** In the TCGA cohort, risk scores were compared within TNM stage and TCGA and CGS subtypes. **h**–**j** In the ACRG cohort, risk scores were compared within TNM stage and ACRG and CGS subtypes. *P* values at the top of each plot were calculated using Kruskal–Wallis test and *P* values over the group pairs were calculated using Wilcoxon test.

We used three external datasets, SNU (*n* = 83), the Cancer Genome Atlas (TCGA, *n* = 151), and the Asian Cancer Research Group (ACRG, *n* = 285), for validating the risk score [3, 4]. The risk score of each cohort enabled us to divide high- (>mean +1 SD), intermediate- (between mean +1 SD and mean −1 SD), and low-risk groups (<mean −1 SD). In the SNU (*P* = 0.03339) and ACRG (*P* = 0.01142) cohorts, the diffuse type of Lauren classification was significantly more frequent in high-risk group than in low-risk group (Supplementary Fig. S8). In all three cohorts, samples with LN metastasis showed significantly higher risk score than those without LN metastasis (*P* = 0.0018 for SNU, *P* = 0.013 for TCGA, and *P* = 0.048 for ACRG) (Supplementary Fig. S9). We further explored the association of our risk score with TNM stage and other molecular subtypes reported in gastric cancer (Fig. 4). The risk score significantly increased as TNM stage increased in the SNU cohort (*P* = 0.0016) (Fig. 4a) and ACRG cohort (*P* = 0.018) (Fig. 4h). In the SNU cohort, there was a significant difference of risk scores among TCGA subtypes, especially with the lowest score for MSI subtype (*P* = 0.00017) (Fig. 4b). A similar significant difference with the lowest score for MSI subtype was also observed in the TCGA cohort (*P* = 0.00037) (Fig. 4f). Consistent findings were observed with the ACRG subtype in both the SNU (*P* = 0.00014) and ACRG cohorts (*P* < 2e−16), showing the lowest score for MSI subtype, while the highest score for EMT subtype (Fig. 4c, i). Compared to the risk score, the correlation of EMT score demonstrated a significant positive correlation in EMT subtype (*P* = 0.019), and that of the MSI score showed a negative correlation in the MSI subtype (*P* = 0.065) (Supplementary Fig. S10) [4]. Similar results were also found when applying two additional molecular subtypes reported in gastric cancer. Applying the consensus genomic subtypes (CGSs) in all three cohorts [20], the risk score was highest in CGS1 and lowest in CGS5, which were associated with EMT and MSI, respectively (*P* = 0.00028 for SNU, *P* = 0.0000012 for TCGA, and *P* < 2e−16 for ACRG) (Fig. 4d, g, j). Lastly, among the alternative splicing (AS) subtypes in SNU cohort [21], mesenchymal subtype (MesS) had a significantly higher risk score than epithelial subtype (EpiS) (*P* = 0.015) (Supplementary Fig. S11). To summarize, the high-risk group was mainly classified into EMT related subtypes and the low-risk group into MSI related subtypes among the other previously established molecular subtypes (Supplementary Fig. S12).

In both the SNU and TCGA cohorts, mutations in six genes did not lead to changes in their gene expression levels (Supplementary Fig. S13). The low-risk group had variants in several genes associated with MSI, such as ATM for SNU cohort (*P* = 0.035) and ARID1A for TCGA cohort (*P* = 0.002) [22, 23], at significantly higher frequencies than the high-risk group.

**Fig. 5:**
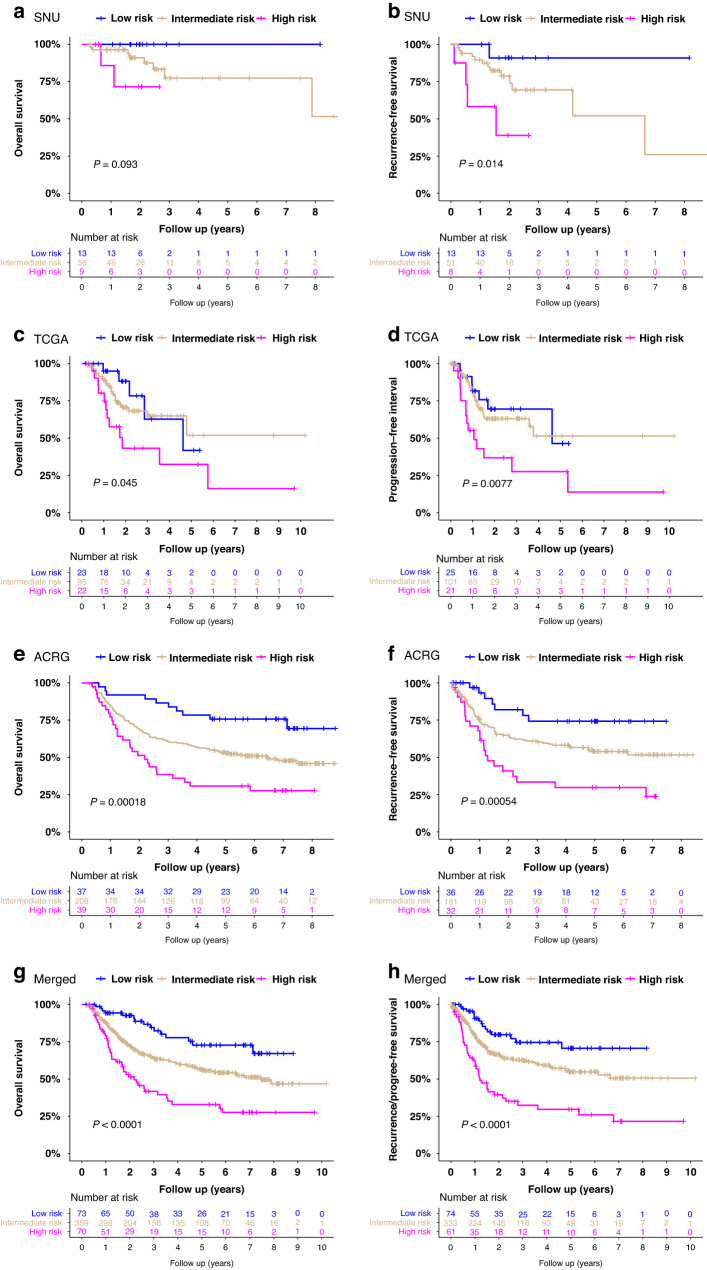
Survival analysis of the risk group in external cohorts. Overall survival (**a**) and recurrence-free survival (**b**) of the SNU cohort. Overall survival (**c**) and progression-free interval (**d**) of the TCGA cohort. Overall survival (**e**) and recurrence-free survival (**f**) of the ACRG cohorts. Overall survival (**g**) and recurrence-free survival (**h**) of a merged cohort including the SNU, the TCGA, and the ACRG cohorts. With mean and standard deviation (SD) of the risk score, risk group was classified as high (>mean +1 SD), intermediate (between mean +1 SD and mean −1 SD), and low risk group (<mean −1 SD) in each cohort.

**Fig. 6:**
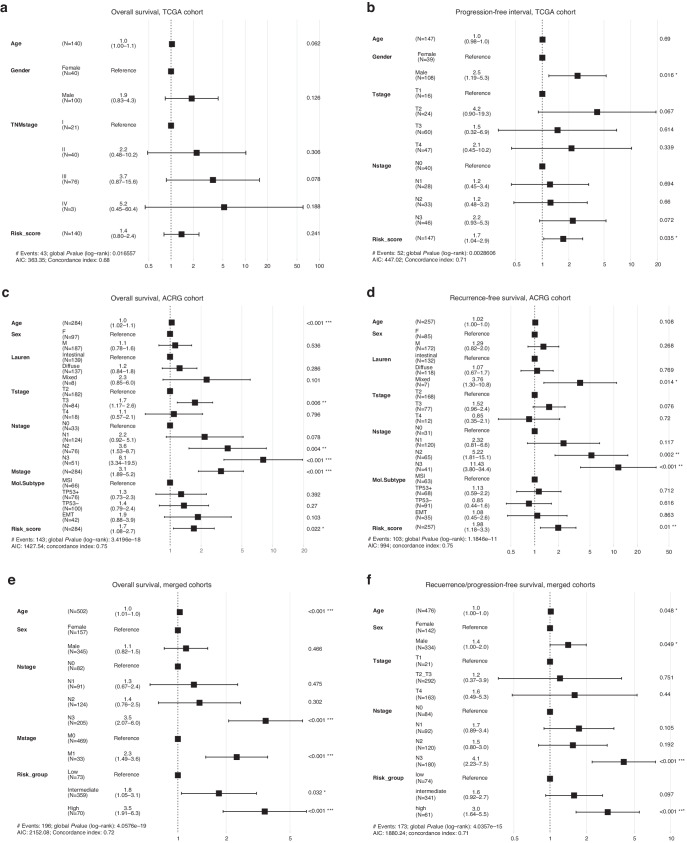
Cox proportional hazard model for external cohorts. Overall survival (**a**) and progression-free interval (**b**) of the TCGA cohort. Overall survival (**c**) and recurrence-free survival (**d**) of the ACRG cohort. Overall survival (**e**) and recurrence-/progression-free survival (**f**) of a merged cohort including the SNU, the TCGA and the ACRG cohorts. With mean and standard deviation (SD) of the risk score, risk group was classified as high (>mean +1 SD), intermediate (between mean +1 SD and mean −1 SD), and low risk group (<mean −1 SD) in each cohort.

Regarding the prognostic implication of risk score, our risk group demonstrated significantly different overall survival in the TCGA (*P* = 0.045) and ACRG (*P* = 0.00018) cohorts and significantly different recurrence- or progression-free survival in all SNU (*P* = 0.014), TCGA (*P* = 0.0077), and ACRG (*P* = 0.00054) cohorts (Fig. 5). A merged cohort consisting of all three external cohorts also showed significantly different overall and recurrence-free survival (both *P* < 0.0001). Cox proportional hazard model revealed that the risk score was an independent prognostic marker for progression-free survival (hazard ratio (HR) = 1.7, *P* = 0.035) in the TCGA cohort, and both overall (HR = 1.70, *P* = 0.022) and recurrence-free survival (HR = 1.98, *P* = 0.01) in the ACRG cohort (Fig. 6). The significance of our risk score outperformed ACRG subtypes for both overall and recurrence-free survival in the ACRG cohort. The high-risk group showed a significantly increased risk of death or recurrence by 3.0 to 3.5 times the low-risk group in a merged cohort (*P* < 0.0001).

## Discussion

Our study demonstrated that the risk score from the 6-gene classifier can successfully predict the prognosis of gastric cancer. It is noteworthy that our classifier was designed from the very early stage tumors all matched with clinicopathologic characteristics but only different metastatic potential. Besides, the risk score can be consistently used as the independent prognostic marker across all TNM stages regarding both overall and recurrence-free survival of different gastric cancer cohorts irrespective of expression platform. Our prognostic model was validated through three different cohorts consisting of two Asian (SNU and ACRG) and one world-wide cohort (TCGA). The prognostic difference of gastric cancer by ethnic disparity has been long-standing controversial issue, and that the TCGA cohort also failed to show discrete prognosis differences based on their four subtypes [3, 24]. The risk score calculated by our 6-gene classifier successfully classified gastric cancer into different groups with statistically different prognoses irrespective of that ethnic disparity, and outperformed the previous classification from the ACRG cohort in the multivariate hazard model.

Our in vitro experiments using cell lines explained the survival difference by increased invasion potential and the resistance against the current 1^st^ line chemotherapeutic regimen of 5-FU and oxaliplatin [11, 25]. A previous study analyzing the chemotherapy response for resectable advanced gastric cancer showed that no-benefit group against 5-FU and oxaliplatin accounted for 55% (344/625) [26]. Based on our 6-gene classifier, a pan-histone deacetylase inhibitor was tested on cell lines engineered to high-risk score, and successfully maintained or increased drug sensitivity, unlike traditional 5-FU and oxaliplatin. As the enzyme plays a role in cancer development, overexpression of HDAC can lead to tumor progression by deacetylating lysine residues in histones and increasing chromatin’s condensation. This process can decrease tumor suppressor gene expression or intrinsic resistance to DNA targeting drugs, and activate cell-cycle associated proteins [27]. Retrospective analysis of high HDAC expression reported a significant association with nodal spread as an independent prognostic marker for gastric cancer [28]. In addition, clinical trials using HDAC inhibitors for anticancer therapy have also remarkably increased, mainly for hematologic malignancy. A recent phase III randomized clinical trial using HDAC inhibitor demonstrated improved survival for advanced breast cancer [29, 30]. Based on our results, a future clinical trial using HDAC inhibitor would provide promising evidence for treating advanced gastric cancers with high-risk scores or resistance to traditional 5-FU or oxaliplatin.

NPM1, Nucleophosmin, is a multifunctional protein that plays a crucial role in maintaining nucleolar structure, cell cycle progression, and histone assembly [31–33]. Overexpression of NPM1 often correlates with mitotic index, metastasis, ribosome biogenesis, or protein synthesis amplified in various solid tumors [34–38]. Although mechanism of NPM1 to cancer progression is still controversial, in vitro study reported that overexpression of NPM1 induced S-phase population in p53-negative cells [39]. In addition to the inhibition of TP53 by the ARF-MDM2-TP53 pathway, overexpression of NPM1 induces cellular growth and proliferation in a dose-dependent manner, suggesting NPM1 as a biomarker for cancer growth [40–42]. Besides, several recent studies have suggested NPM1 as a good target for targeted cancer therapy [38, 43, 44]. Even though our study showed the possibility of HDAC inhibitor for patients in the high-risk group, inhibition of NPM1 based on not only the direct effect on NPM1 itself but also indirect effects including sensitizing cancer cells would be the promising treatment strategy in the future [38].

DTX3 was reported as one of the eight essential genes for cell proliferation in luminal-subtype breast cancer according to an integrated genomic approach [45]. Overexpression of DTX3 also induced ovarian cancer cell growth and invasion in a mutant P53-dependent fashion by reducing MDM2-p53 binding [46]. Since TP53 is one of the most potent tumor suppressor genes, expression change of one gene related to TP53 may not be enough to affect the tumor suppressive function. To the best of our knowledge, our study could be the first report showing the role of DTX3 in gastric cancer progression in conjunction with other TP53 linked genes (HDAC and NPM1).

MED12 gene is noted to have copy number alterations or somatic mutations, or aberrant expressions in various cancers, but the prognostic significance of these changes is not clear [47]. Consistent with our results, MED12 loss could induce an EMT-like phenotype through activation of TGF-βR signaling pathway, which was associated with resistance to chemotherapy such as 5-FU and cisplatin in colon cancer patients [48]. The RAS-RAF-MEK-ERK pathway (downstream of EGFR) could be activated by a low MED12 induced TGF-βR signaling pathway, suggesting that the EGFR Inhibitor would not be effective for advanced gastric cancer with a high-risk score. The downregulation of MED12 might explain why EGFR Inhibitor therapy was ineffective in previous clinical trials for advanced gastric cancer [47, 49–51].

PPP3R1, the regulatory subunit B of calcineurin, is a well-known target of the immunosuppressant drug-receptor complexes. Calcineurin regulates critical biological processes such as T cell immune response and cell cycle control [52]. A previous in vitro study revealed that calcineurin and NFAT factors are constitutively expressed by primary intestinal epithelial cells, and selectively activated in intestinal tumors due to impaired stratification of the tumor-associated microbiota and toll-like receptor signaling, which eventually promotes tumor proliferation and prevents apoptosis [53]. As one of immune related gene signatures, overexpression of PPP3R1 was also associated with poor prognosis of colorectal cancer patients [54]. Considering the recent dramatic evolution of immunotherapy for gastric cancer, whose background is one of the most pro-inflammatory microenvironments among gastrointestinal cancers, we hope our classification will help identify the appropriate subset of gastric cancer patients for immunotherapy.

Taken together, six genes closely contribute to the progression of gastric cancer, particularly growth, cell cycle, and resistance to cytotoxic chemotherapy, albeit in different directions in expression. The poor prognosis is presumably based on EMT-driven cancer progression, as the gastric cancer patients classified as high-risk group have no shared mutations and mainly belong to EMT-related subtypes across all different molecular classifications of gastric cancer.

Early gastric cancers invading only the mucosal layer with LN metastasis are precious and need more than a decade to collect. Even though we customized several genes through the extensive literature review, the genes of the PanCancer panel in our study included only 800 genes. Considering long storage and small volume of samples, rather than usual RNA sequencing, NanoString nCounter assay was considered to provide more reproducible results at very low input of RNA without amplification process [55]. Therefore, instead of using an assay that is not robust and likely to produce false positive results, we decided to use the Nanostring platform with robust expression quantitation even though we do provide a genome-wide expression profile. To the best of our knowledge, this is the first study analyzing cancers with clinically different progression feature in the earliest stage whose baseline clinicopathologic characteristics were statistically matched to the control group.

In conclusion, the machine learning model of the 6-gene classifier consisting of HDAC5, NPM1, DTX3, MED12, TP53, and PPP3R1 can successfully predicts the prognosis of gastric cancer across all TNM stages in three different gastric cancer cohorts worldwide, irrespective of expression platform.

## Materials and methods

### Sample preparation

We identified 18 early gastric cancer samples with lymph node metastasis (Npos) out of 1003 early gastric cancer samples which were reported in our previous study [15]. To select the control group as early gastric cancer without lymph node metastasis (Nneg) in remaining 985 samples, we carried out 1:1 propensity score (PS) matching by which exact matching was conducted for differentiation, and PS nearest neighbor matching for age, sex, location, tumor size, and Lauren classification using SPSS version 21.0 (SPSS, Inc., Chicago, IL, USA). Formalin-fixed paraffin-embedded (FFPE) samples from corresponding surgical specimens were identified through the repository of the Department of Pathology, Seoul National University Hospital. Tumor, normal mucosa, and metastatic lymph node lesions were microdissected from sections with 10 μm thickness of FFPE samples. Microdissection was conducted using hematoxylin and eosin-stained slides with needle and blade under the microscope by the expert pathologist (WHK). The study protocol was approved by the Institutional Review Board of Seoul National University Hospital (IRB No: H-1708-166-882) and Seoul National University Hospital Bundang Hospital (IRB No: B-2006-621-305).

### NanoString assay

Total RNA was then extracted using Lucigen-Epicentre MasterPure Complete DNA/RNA Purification Kit. For extracted RNA, yield and purity of were assessed using a DS 11 Spectrophotometer (Denovix Inc, DE, USA) and the quality was checked using Fragment Analyzer(Advanced Analytical Technologies, IA, USA). Considering severe fragmentation of RNA in FFPE sample, 1 μg of total RNA with the concentration of 1 μg/5 μl per sample was used for NanoString assay. NanoString assay was conducted using the customized nCounter PanCancer Pathways panel including default 770 genes plus 30 manually chosen genes which were selected based on literature review during the recent 5 years at the time of panel customization (Supplementary Table S5).

For each assay, a high-density scan encompassing 555 fields of view was performed, and the final data were collected using the nCounter Digital Analyzer. Quality control (QC) of nCounter data was conducted using NanoString nSolver Analysis Software v4.0. For Imaging QC, at least 75% of fields of view should be successfully counted to obtain robust data. For binding density QC, the range of 0.1 and 2.25 spots per square micron was established for assays. For positive control linearity QC, correlation values between the known concentrations of positive control target molecules added by Nanostring and the resulting counts were ≥0.95. For positive control limit of detection QC, the counts for the 0.5fM positive control probe was higher than background which was represented by the 2 standard deviations above the mean of the expression of negative control probes. Samples for downstream analysis passed all QC criteria.

Initial normalization of nCounter data was conducted by NanoStringNorm 1.2.1, R package [56]. For more confident housekeeping genes, 29 genes with *P*-value ≥ 0.01 or Pearson’s r < 0.8 in housekeeping genes in nCounter data were excluded before normalization. Normalization methods for NanoStringNorm were selected as the methods with the lowest coefficient of variation for control genes which were “geo.mean” for code count, “mean” for background, and “housekeeping.sum” for sample content.

Differential expression was analyzed by DESeq2 1.24.0, R package using normalized nCounter data [57]. All other downstream analyses were conducted after the variance stabilizing transformation of expression data by DESeq2. Canonical pathway analysis was performed by Ingenuity Pathway analysis (QIAGEN Inc.). Analysis database was used as signaling pathways including apoptosis, cell cycle regulation, cellular growth proliferation and development, cytokine signaling, growth factor signaling, intracellular and second messenger signaling, nuclear receptor signaling, organismal growth and development, and transcriptional regulation.

### Prediction model and risk score

For feature selection for more accurate prediction, we used Lasso ${l}_{1}$ penalization with the sparse Partial Least Squares Discriminant Analysis (sPLS-DA) from mixOmics 6.8.0, R package [16]. The number of optimal PLS components and selected features in each component was tested toward the lowest balanced error rate with 5-fold cross-validation. With that optimal components and features, the performance of sPLS-DA model was tested with leave-one-out cross-validation (LOOCV). For more consistent prognostic classifier, we analyzed Spearman correlation for those selected features, and any genes with *P* ≥ 0.001 were excluded sequentially until all remained genes were significantly correlated with *P* < 0.001. After a thorough review for the functional implication of those correlated genes, the final genes were selected as the signature classifier to predict poor prognosis of gastric cancer. Classification efficacy of the signature classifier was tested with sPLS-DA or Random Forest model (randomForest 4.6–14, R package) with LOOCV. As parameters in Random Forest, the number of trees used was chosen as 10,000, and the number of variables randomly sampled as candidates at each split was decided as 2 with respect to the lowest out-of-bag (OOB) error estimate, by which OOB error could not be improved by 1e−5 or more. Variable importance was retrieved by “varImp” function of the caret package in R from Random Forest model including the final classifier. Considering the direction of fold change between Npos and Nneg, we multiplied the variable importance of TP53 and MED12 by −1. The risk score per sample was calculated as the sum of the expression of genes in the final classifier weighted by each variable importance. Using the mean and SD of the risk score within each cohort, we divided high- (>mean + SD), intermediate- (between mean + SD and mean − SD), and low-risk groups (<mean − SD).

### Cell culture, gene knockout using CRISPR/cas9, and overexpression

MKN-1, MKN-74, and SNU-216 cells were obtained from the Korean Cell Line Bank (KCLB, Seoul, Korea). All cells were certified KCLB and mycoplasma testing was routinely performed using e-MycoTM plus Mycoplasma PCR Detection Kit (Intron, Korea), verifying that the cells were mycoplasma free. Cells were maintained in PRMI1640 medium (Gibco, Thermo Fisher Scientific) containing 10% fetal bovine serum (Gibco) and 1% Penicillin streptomycin (Gibco) and maintained in a humidified incubator with 5% CO_2_ at 37 °C. TP53 and MED12 sgRNA sequence referred the human GeCKO lentiviral pooled library [58] sequence (Supplementary Table S6). Lentiviral target plasmid were cloning with lentiCRISPRv2 backbone (Addgene #52961) and co-transfected with lentiviral helper plasmid pCMV-VSV-G (Addgene #8454) and psPAX2 (Addgene #12260) in 293FT cells using Lipofectamine 2000 (Life Technologies). Lentiviral supernatants was concentrated with Lenti-X concentrator (Clontech Laboratories, Inc.) according to the manufacturer’s protocol. TP53 and MED12 gene knockout in gastric cancer cells confirmed with western blot. The expression plasmid for GFP-NPM1 (Addgene # 17578) and Flag-HDAC5 (Addgen # 13822) were purchased from Addgene. The vectors containing cDNAs encoding Flag-PPP3R1 and Flag-DTX3 were cloning with pCMV-tag2B vector (Agilent Technologies). Plasmid transfection were performed using the Neon transfection system (Thermo Fisher Scientific) into control or TP53 and MED12 knockout gastric cancer cells following the manufacturer’s protocol. After 48 h, the transfected cells were subjected to the following in vitro assays.

MKN-1 sgNC cells stably expressing luciferase (MKN-1-sgNC-Luc) and MKN-1 TP53/MED12 double knockout cells stably expressing luciferase (MKN-1-KO-Luc) were established by lentivirus infection of pLenti CMV/TO V5-Luc Puro (w549-1) (addgene # 19785) then transfected pCMV-tag2B vector for control (MKN-1-sgNC-Luc-vec) or GFP-NPM1, Flag-HDAC5, Flag-PPP3R1 and Flag-DTX3 plasmid for 4 gene overexpression (MKN-1-KO-Luc-OE). After 48 h, cells were detached with TripleLE and washed twice with PBS and resuspension in PBS.

### In vivo mouse experiment

All animal experiments conformed to the Institutional Animal Care and Use Committee (IACUC) guideline and were approved by the Animal Research Committee of Seoul National University Bundang Hospital (IACUC number: BA-2311-379-001). Five-week-old female nude mice (BULB/cSlcnu/nu) were obtained from OrientBio (Seongnam, Korea). Mice were housed and adapted to the breeding environment for one week before the experiment. A total of 5 × 10^5^ MKN-1-sgNC-Luc-vec or MKN-1-KO-Luc-OE cells were suspended 100 $\mu \ell$ of PBS and injected into the tail vein of nude mice. To visualize the metastatic tumors, mice were intraperitoneally injected with 150 mg/kg VivoGlo^TM^ Luciferin (#P1043, Promega) every week and photonic emission was imaged using the In Vivo Imaging System (IVIS, Perkin Elmer) Lumina II with a collection time of 1 min. Luminescent activity in the region of interest (ROI) was quantified by integrating the photonic flux (photons per second) through a region encircling each tumor as determined by the LIVING IMAGES software package per manufacturer’s instructions (Perkin Elmer). *P* value of ROI was calculated using unpaired t-test with two-tailed by Graphpad Prism 9.5. Three weeks after cell transplantation, all mice were sacrificed and each organ was autopsied. Tissues were fixed in 10% neutral buffered formalin and embedded in paraffin. Then, tissues were sectioned into 4 μm thickness. The slides were subjected to hematoxylin and eosin (H&E) with BenchMark ULTRA IHC/ISH System (Roche). The slide images were evaluated by a pathologist.

### Western blot and quantitative real-time PCR (qPCR)

Cells were lysed in RIPA buffer (Thermo Scientific) containing protease inhibitor cocktail (Roche) and phosphatase inhibitor cocktail (Roche), and were centrifuged at 13,000 × *g* for 10 min at 4 °C. After determination of protein concentration in the cell extract by the BCA method (Thermo Scientific), 20 ug of protein were resolved by SDS-PAGE and transferred to polyvinyl difluoride membrane. Membranes were blocked for 30 min with blocking buffer (Bio-Rad) and incubated with following antibody; TP53 (Cell Signaling #2527), MED12 (Cell Signaling #4529), anti-Flag (Sigma-Aldrich Corporation; F1804), anti-GFP(Invitrogen; MA5-15256), and beta-actin(Sigma-Aldrich A1978) antibody. The membranes were washed and incubated with horseradish peroxidase-conjugated secondary antibody, followed by enhanced chemiluminescence development according to the manufacturer’s instructions.

Total RNA extraction was performed Qiagen RNeasy plus mini kit according to the manufacturer’s protocol (Qiagen). The quantity of RNA was measured by Nanodrop 1000 (Thermo Scientific). Two micrograms of total RNA was reverse transcribed with Superscript III transcriptase (Invitrogen). qPCR was performed by using SYBR Green Master Mix (Applied Biosystems) in QuantStudio 7 Real-Time PCR system (Applied Biosystems) with 10 ng cDNAs as templates in each reaction. qPCR analyses were performed by relative quantification method normalized with GAPDH. The sequence of the qPCR primer pairs are shown in Supplementary Table S7.

### In vitro migration, invasion, wound healing, and cytotoxicity assay

Migration and invasion assays were performed using 8.0-µm pore inserts in a 24-well Transwell (BD Biosciences). Transfected cells were added to the upper chamber of a transwell (5 ×10^3^ cells per well) with a non-coated filter and incubated for 48 h in the migration assay. The invasion assays were performed using 12.5% Matrigel (Corning)-coated filters at 5 ×10^3^ cells per well, and the cells were incubated for 72 h. The migrated or invaded cells were fixed with 70% ethanol then stained with 0.4% Crystal violet solution. For the wound measuring, a scratch on complete confluence was made, and the percentage of cell-free area at 24 h was measured relative to the distance at 0 h (100%) using photographed images. Each experiment was performed in triplicates and the mean values were presented.

For the cell viability assays, approximately 3000 cells were plated in each well of a 96-well plate and incubated at 37 °C with 5% CO_2_ for 1 day then added with the indicated drugs in triplicate at serially diluted concentrations with 100ul medium, respectively. Cells were treated with the following reagents at the indicated final concentration: 5-FU (1 mM), Oxaliplatin (250 µM), and Panobinostat (1 µM) for 72 h and examined for cell viability using the EzCytox WST assay kit (Daeil Lab, Korea). Cell viabilities were estimated as relative values compared to the untreated controls.

### External datasets: SNU, TCGA, and ACRG cohort

For SNU cohort, we used next-generation sequencing data retrieved from the snap fresh frozen tissue repository between 2001 and 2015 at the lab of gastric cancer biology, Cancer Research Institute, SNU. All RNA samples extracted from the SNU cohort were processed for the mRNA-focused sequencing library using the Illumina TruSeq RNA Sample Prep Kit v2. The paired-end reads (2 ×101 bp) were sequenced on an Illumina HiSeq2000 platform (Illumina Inc., San Diego, CA). Read alignment was performed using STAR aligner 2.6.1.d (2-pass mode) with the *Homo sapiens* GRCh38 Ensembl v94 gene primary assembly [59]. The mRNA expression was quantified for downstream analysis by expected read count based on effective gene length using RNA-Seq by Expectation-Maximization (RSEM 1.3.1) [60]. The quantified mRNA expression was analyzed for DEGs by DESeq2, and variance stabilizing transformation was used for downstream analysis [57].

Whole-exome sequencing (WES) of the dsDNA from tumor and corresponding normal gastric mucosa samples was performed using the Agilent SureSelect Human All Exon V5 + UTR region kit (Agilent Inc., Santa Clara, CA, USA). The paired-end reads (2 ×101 bp) were sequenced on an Illumina HiSeq2000 platform (Illumina Inc., San Diego, CA). Based on the *Homo sapiens* GRCh38 Ensembl v94 gene primary assembly, the read alignment, deduplication and base recalibration processing were performed using the Burrows–Wheeler Aligner (bwakit-0.7.15) and Picard in Genome Analysis Toolkit 4.1.0.0 (GATK4), following the recommended best practices [61–63]. The somatic mutations were called by mutect2 in GATK4 with aligned whole-exome sequencing data [64]. Confident somatic calls were determined as the passed variants after filtering the cross-sample contamination calculated by FilterMutectCalls and the OxoG artifacts calculated by FilterByOrientationBias in GATK4. The functional annotation of variants was performed with ANNOVAR 2018Apr16 [65]. Variants with 1) 10 or more total read depths for the normal allele, 2) 20 or more read depth for the tumor allele, and 3) 5% or more alternative allele fraction were selected. Only variants with population frequencies of <0.01 in the overall population as determined by Genome Aggregation Database were included [66]. The Fisher’s exact test was performed between the groups for genes that were mutated by more than 40% in high- or low-risk group and our six genes, and visualized using Maftools 2.16.0, R package [67].

Sequencing data is archived in Gene Expression Omnibus (https://www.ncbi.nlm.nih.gov/geo/query/acc.cgi, accession number: GSE126304), Sequence Read Archive (https://www.ncbi.nlm.nih.gov/sra/, accession number: PRJNA521397), and our clinical cancer genome database (http://ccgd.snu.ac.kr/index.html).

For pathological microsatellite instability (MSI), fragment analysis was used to compare the tumor and normal tissue samples at 5 base pair (bp) locations after polymerase chain reaction using the following two primers: Primer 1 consisted of BAT26 (116 bp) and BAT25 (148 bp), and primer 2 consisted of D5S346 (96–122 bp), D17S250 (146–165 bp) and D2S123 (144–174 bp).

For the classification ACRG subtypes in the SNU cohort, we calculated the signature scores based on the gene list of each signature including MSI, EMT, and TP53 activity using the same manner used in the previous study (Supplementary Fig. S14) [4]. The cut-off of TP53 signature score (11.89059 in log2 scale) was identified as Youden index of the Receiver Operating Characteristic curve between TP53 signature score and the somatic mutation of TP53 gene. A web-based GPICS120 predictor was used for CGSs classification (https://kasaha1.shinyapps.io/GPICS120) [68]. The classification of TCGA and AS subtypes for SNU cohort was conducted and described in our previous study [69]. They were classified using approximately 800 signature gene classifiers and splicing events of eight genes, respectively [69, 70].

For TCGA dataset, level 3 mRNA expression data (rnaseqv2_unc) processed by RSEM was downloaded from Broad GDAC Firehose (http://gdac.broadinstitute.org) [71]. Expected read count by RSEM was normalized as variance stabilizing transformation by DESeq2, and subsequently used for the calculation of risk score. Clinical information was retrieved from phenotype data of GDC TCGA stomach cancer cohort in UCSC Xena [72]. Survival data, the information of molecular subtypes, and genetic mutation data were retrieved from TCGA PanCancer data [73]. Regarding molecular subtypes, POLE type in PanCancer data was replaced by each previous subtype originally reported in 2014 [3]. For reliable prognostic analysis, the standardized treatment and accurate pathological information are essential, and any prior systemic treatment may affect RNA expression profile. Therefore, we exclude samples with a minimal number of total examined lymph nodes less than 16, those with TNM stage which cannot be assessed, those with history of prior malignancy, and those with history of any neoadjuvant treatment. Also, we only included samples with R0 status.

For ACRG dataset, we downloaded pre-processed expression data from GEO (GSE62254), and transformed to log2 scale [4]. In terms of gene expression summary, we excluded probes with the name including “_s” or “_x” which may hit different genes. For NPM1, there were only probes with “_s” or “_x”, and the expression level measured by 221691_x_at was reported as not consistent for the composite expression of the RefSeqs of NPM1 [74]. Therefore, we allowed the probes with “_s” for NPM1 in our classifier genes. To summarize probe signal intensity, we chose one representative probe with the maximum median level of expression across all samples. For accurate downstream prognostic analysis, we exclude samples with a minimal number of total examined lymph nodes less than 16.

### Statistical analysis including survival analysis and Cox proportional hazard model

For overall survival, we excluded samples with follow up period of ≤60 days or 2 months to avoid unwanted biased events (Supplementary Table S8). Cox proportional hazard model included age, sex, T stage, N stage, M stage, and risk score. In case of infinite coefficients for stage variable, TNM stage grouping was used instead of T, N, and M stage. ACRG subtypes were included in the Cox model for ACRG cohort since they were reported as a prognostic marker [4]. For progression-free or recurrence-free survival, samples with M1 stage were excluded for Cox model. Age and risk score were fitted as continuous variables, and pathological stages and molecular subtypes were fitted as categorical variables as they were. In terms of the edition for TNM stages in the TCGA cohort, we excluded samples diagnosed by the 4^th^ edition because of incompatible information to other editions. TNM stage data of samples with the 5^th^ and 6^th^ edition were manually converted to data in the 7^th^ edition, based on original pathological reports of those samples downloaded from TCGAbiolinks [75]. For merged external cohorts including the SNU, the TCGA, and the ACRG cohort, stage information was unified in the 7^th^ edition by which T stage was classified as T1, T2/T3, and T4, even though TNM stage group was not available. In case of the infinite coefficient for T stage, TNM stage group was used instead in the TCGA cohort, or only the N and M stage was used in the merged cohort. The SNU cohort could not be fitted to the Cox proportional hazard model with multiple infinite coefficients due to the sample size. Overall statistical comparison of continuous variables including risk score was conducted using Wilcoxon test between two groups or Kruskal–Wallis test for three or more comparison. Other comparison of categorical variables was analyzed by Fisher’s exact test. All data analysis in this study were performed using R software (version 3.6.0; The R Foundation for Statistical Computing, Vienna, Austria) and GraphPad Prism version 9.0 (GraphPad Software, Inc., La Jolla, CA).

### Supplementary information

Supplementary Figures

Supplementary Tables

## Data Availability

The data generated using Nanostring platform in this study are not publicly available due to patient privacy and consent, but are available as a processed form upon reasonable request to the corresponding author.

## Code availability

The code used for the analysis is available upon reasonable request to the corresponding author.
