# Supplementary material for: RNA expression of 6 genes from metastatic mucosal gastric cancer serves as the global prognostic marker for gastric cancer with functional validation
Source: Br J Cancer. 2024 Mar 11;130(9):1571–84. doi: 10.1038/s41416-024-02642-6 (PMC11059174; doi:10.1038/s41416-024-02642-6)

Supplementary Figures

Supplementary Figure S1. Feature selection by spares Partial Least Squares Regression-Discriminant analysis (sPLS-DA). (a) The selection of PLS component. (b) The selection of features per each component. (c) Feature contribution in component 1 to sPLS-DA model. (d) Principal component analysis with component 1 by sPLS-DA model .

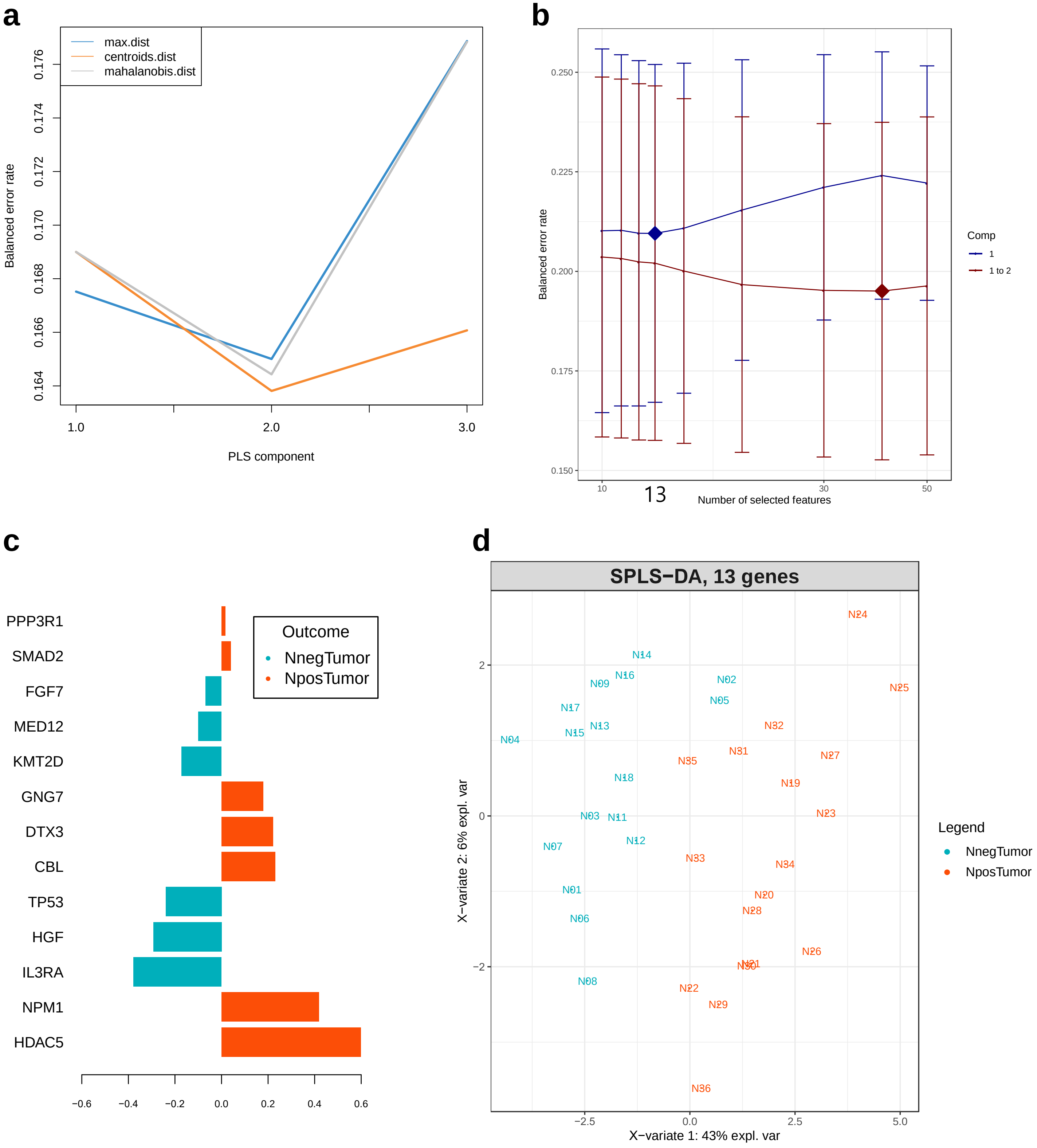

Supplementary Figure S2. Spearman correlation among genes in component 1 of spares Partial Least Squares Regression-Discriminant analysis (sPLS-DA). Any genes with correlation significance of *P*≥ 0.001 were excluded. Size of each circle represents significance of correlation.

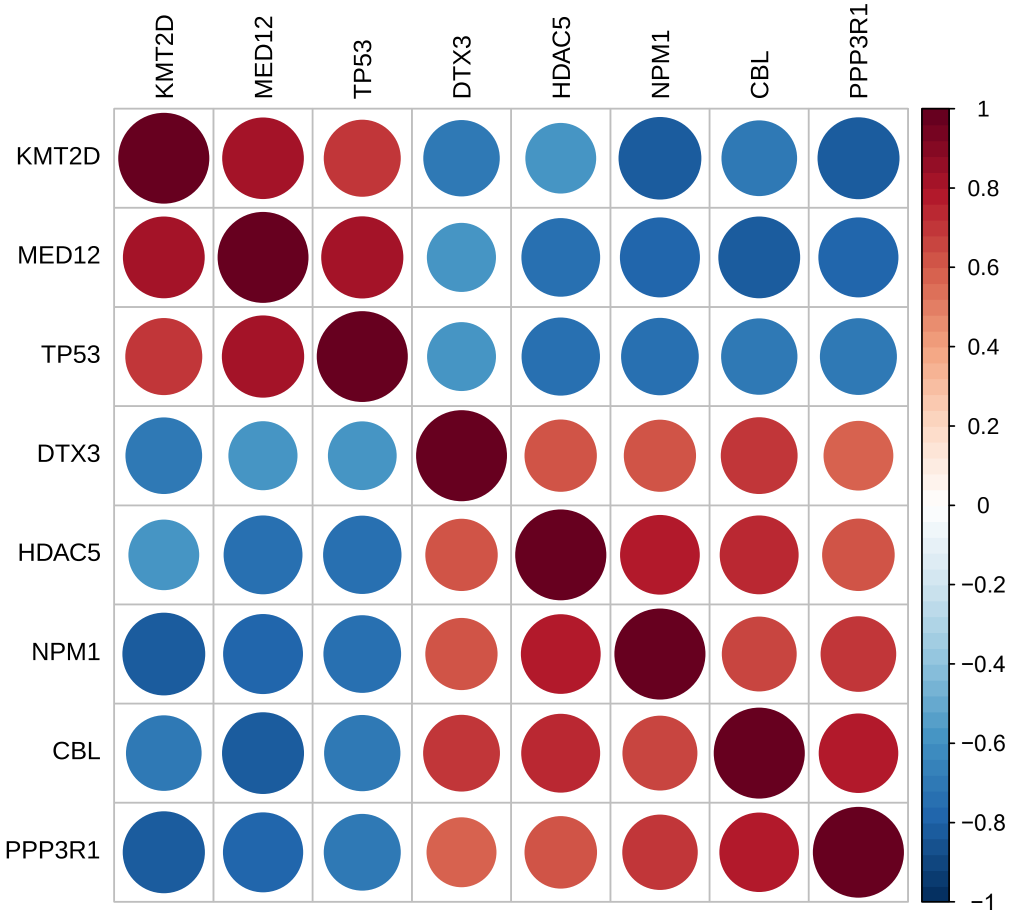

Supplementary Figure S3. Distribution of normalized RNA expression and the risk score of different datasets. Distribution of overall expression (a) and risk score (b) of NanoString dataset (training set), overall read count (c) and risk score (d) of the SNU cohort, overall read count (e) and risk score (f) of the TCGA cohort, and overall probe signal intensity (g) and risk score (h) in the ACRG cohort. Blue dotted line represents mean value of overall expression in each cohort, red dotted line is -1 and +1 standard deviation from mean value of risk score in each dataset.

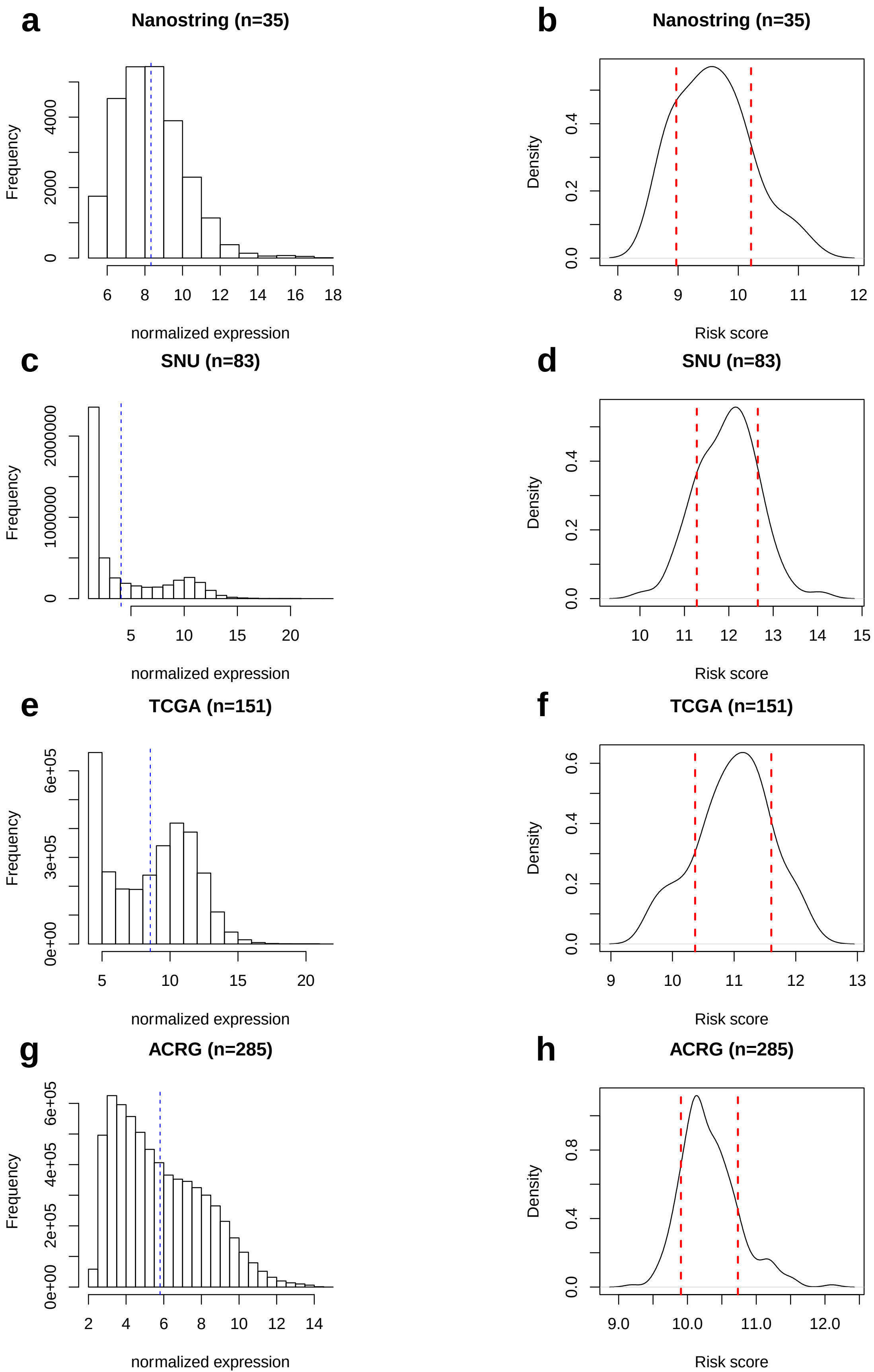

Supplementary Figure S4. Distribution of the risk score from 6 genes in 37 gastric cancer cell lines. We selected MKN-74 (low risk score), SNU-216 (middle risk score) and MKN-1 (high risk score) cell line for in vitro experiments

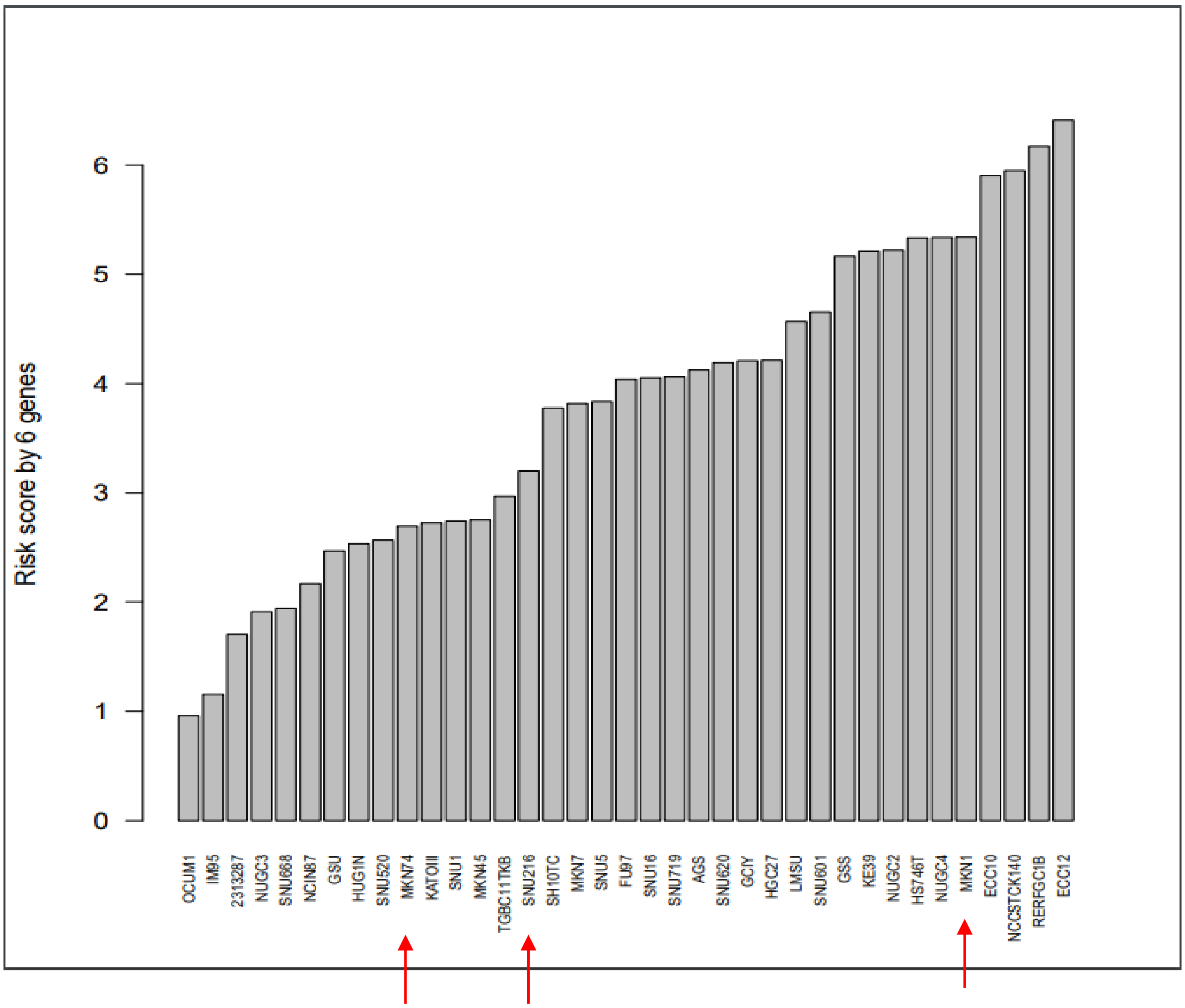

Supplementary Figure S5. TP53/MED12 DKO and HDAC5, NPM1, DTX3 and PPP3R1 co-expression enhance the migration and invasion of GC cells. The effect of 6 gene expression on the migration and invasion ability of GC cells (MKN-74, SNU-216 and MKN-1) was determined using transwell chamber. All experiments were performed in triplicated, and the red arrow means migrated or invaded cell. sgNC-Vec: the control vector transfection in the negative control sgRNA infected cell; sgNC-OE: HDAC5, NPM1, DTX3 and PPP3R1 co-transfection in the negative control sgRNA infected cell; KO-Vec: the control vector transfection in TP53/MED12 DKO cell; KO-OE: HDAC5, NPM1, DTX3 and PPP3R1 co-transfection in TP53/MED12 DKO cells.

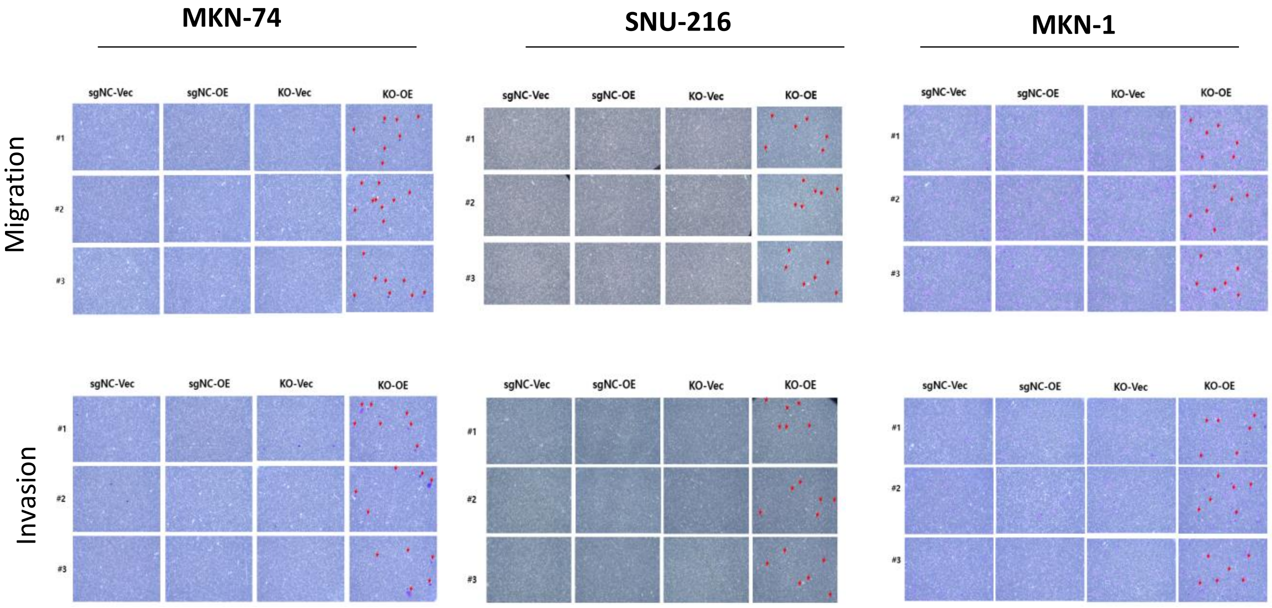

Supplementary Figure S6. Effect of chemotherapeutic drugs 5-FU, Oxaliplatin and Panobinostat in TP53/MED12 DKO and HDAC5, NPM1, DTX3 and PPP3R1 co-expressed MKN-74, SNU-216 and MKN-1 cells. Increasing expression of HDAC5, NPM1, DTX3, and PPP3R1, and decreasing of TP53 and MED12 shows a resistance effect with 5-FU or Oxaliplatin in MKN-74 and MKN1 cells. Panobinostat significantly decreased in KO-OE SNU-216 cells compare with each control cells. Data are represented as mean values ± standard error of mean (SEM) based on triplicate experiments, and the regression lines were calculated by dose-response inhibition models.

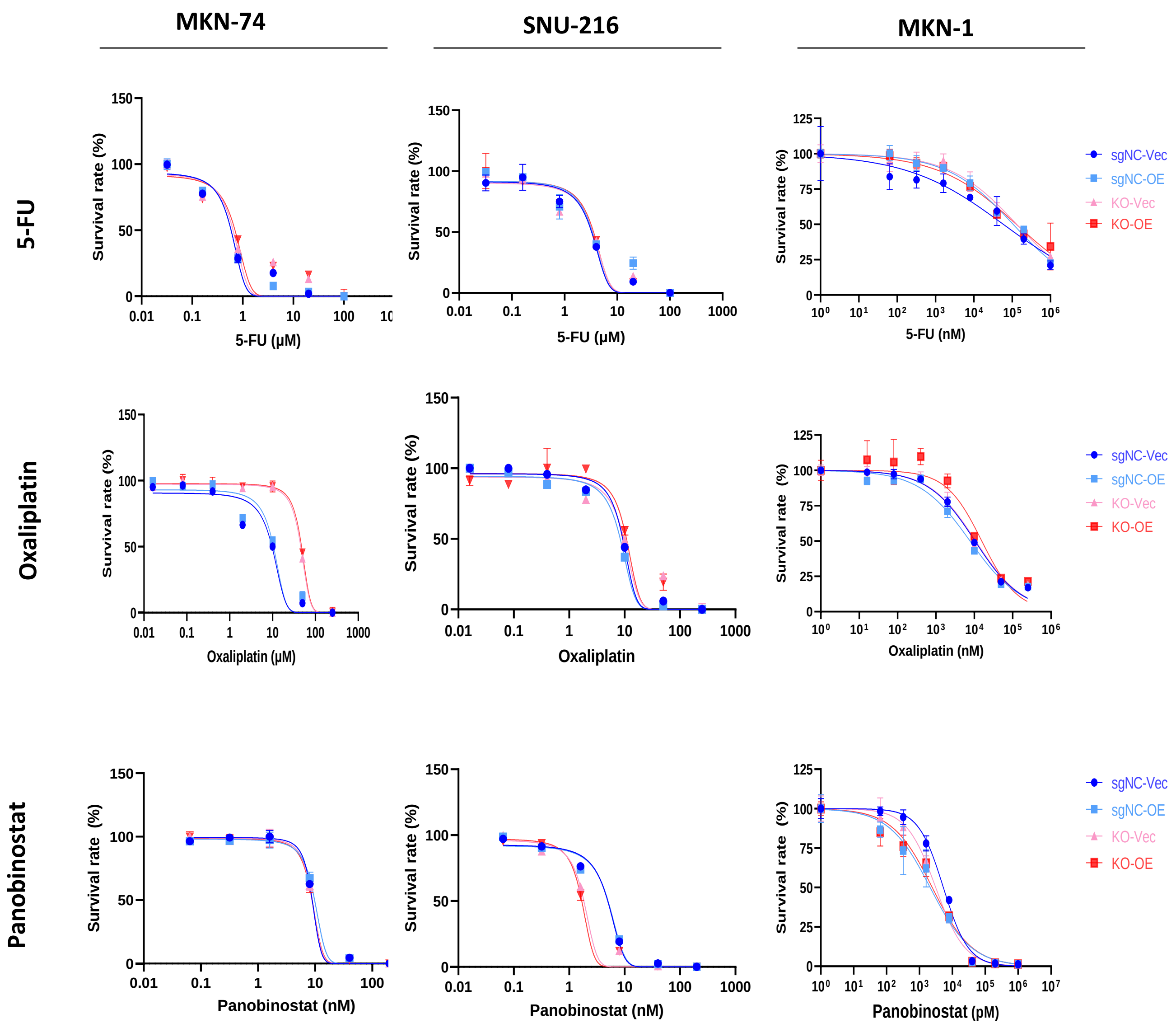

Supplementary Figure S7. Hematoxylin and eosin (H&E) staining image of MKN-1-sgNC-Luc-Vec (control) or MKN-1-KO-Luc-OE (KO-OE line) injected mouse lung, liver and intestine. Black arrows indicate engrafted human cancer cells (scale bar: 100μm).

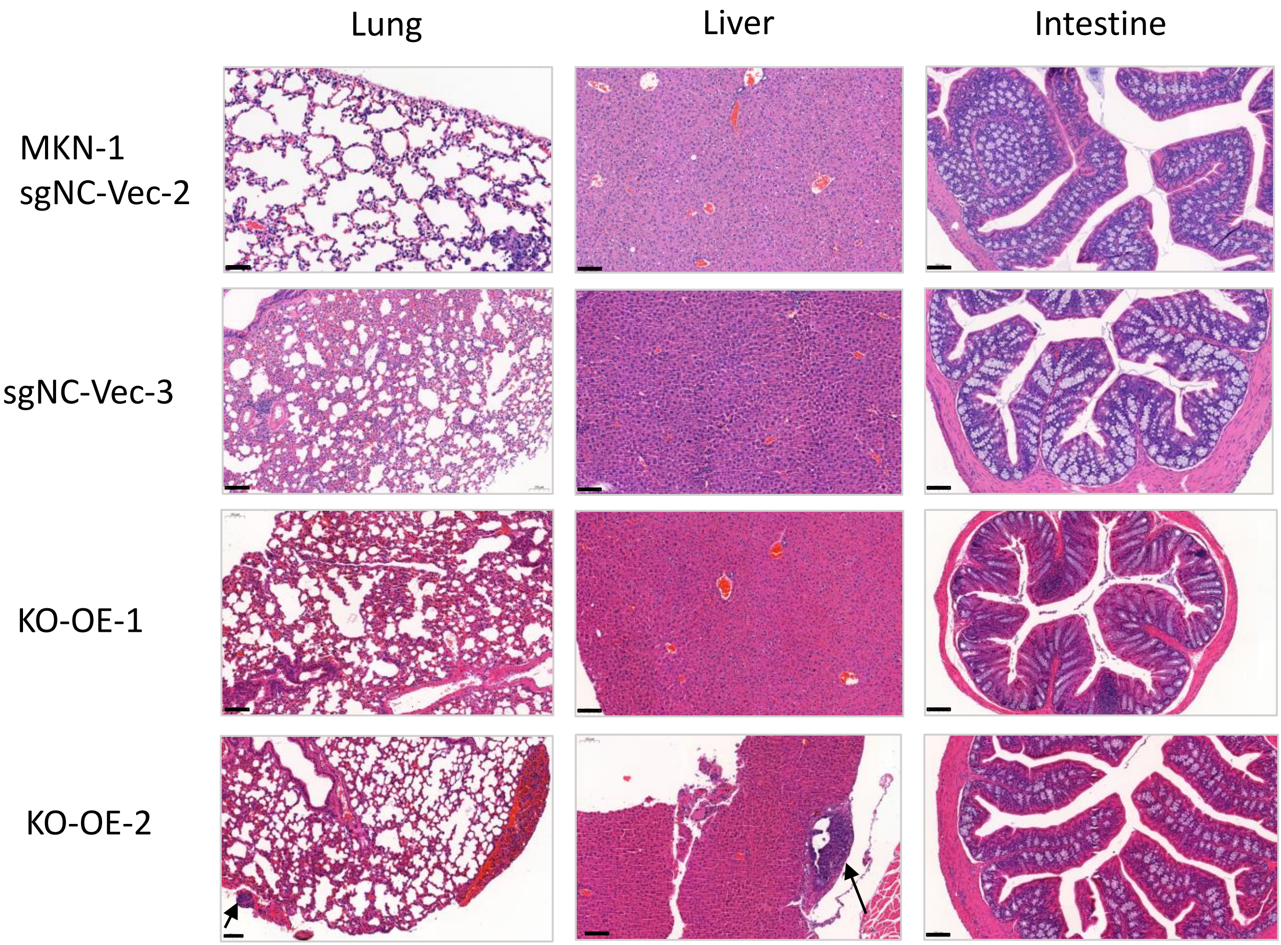

Supplementary Figure S8. Expression of 6 classifier genes with risk score in each external cohorts. (a) the SNU cohort. (b) the TCGA cohort. (c) the ACRG cohort. With mean and standard deviation (SD) of risk scores in each cohort, group was classified as high (>mean+SD), intermediate (between mean+SD and mean–SD), and low risk group (<mean–SD). *P* value was calculated between high- and low-risk group with Fisher's exact test.

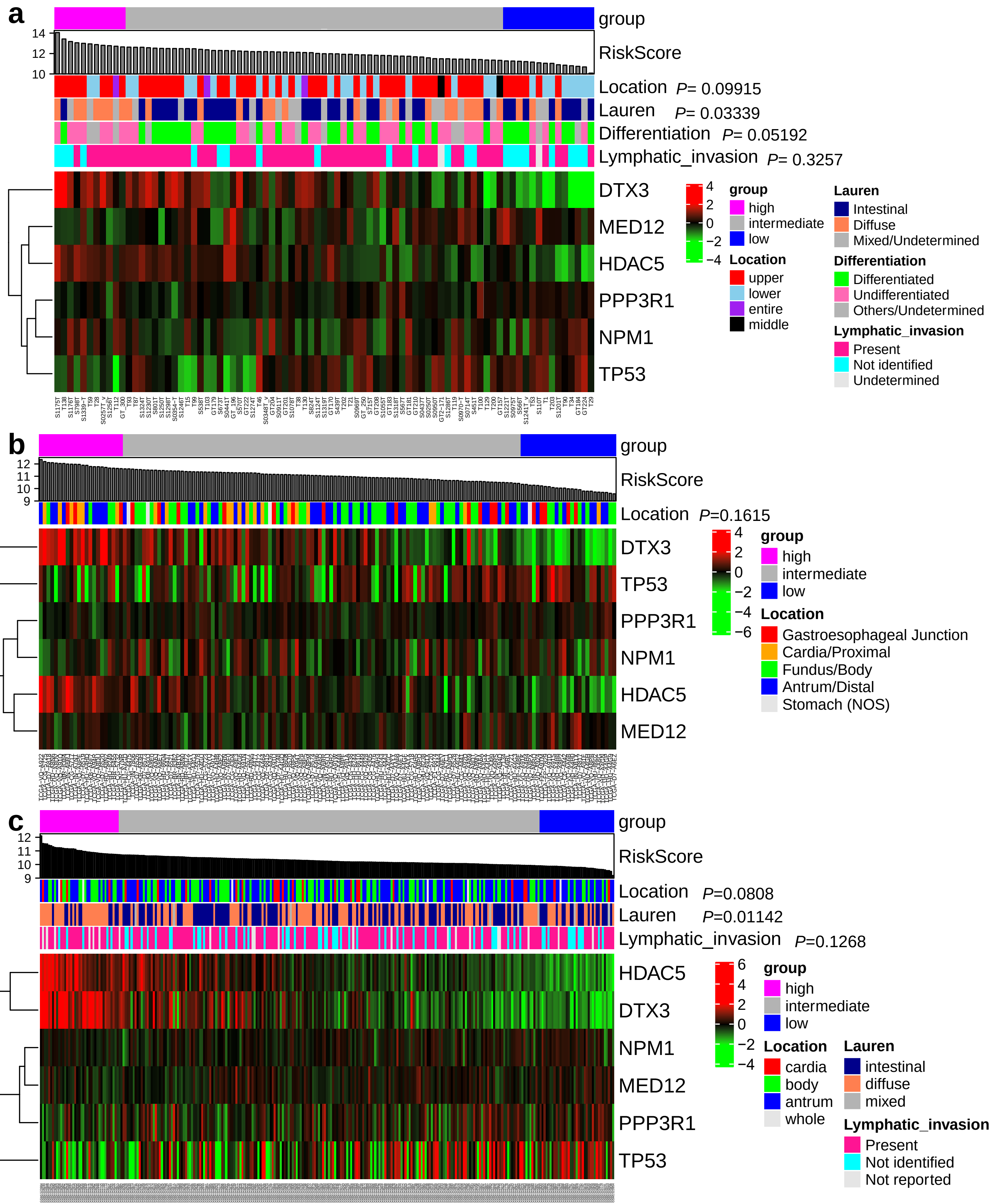

Supplementary Figure S9. Risk scores between samples with LN metastasis and those without LN metastasis. (a) the SNU cohort. (b) the TCGA cohort. (c) the ACRG cohort.

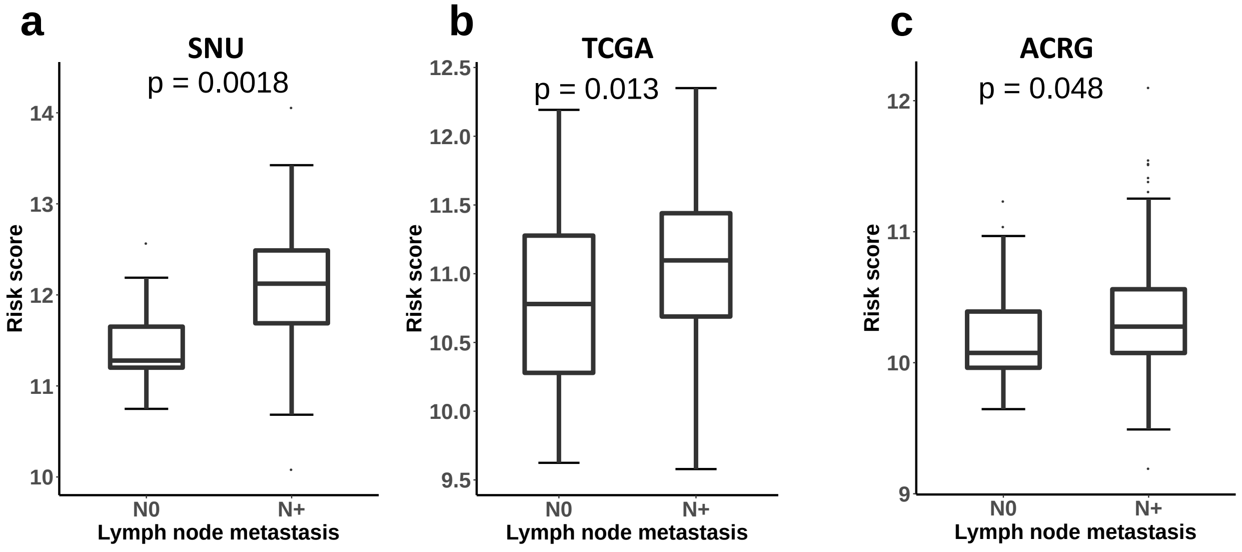

Supplementary Figure S10. The Pearson correlation between epithelial-mesenchymal transition (EMT), microsatellite instability (MSI) scores and risk scores in the SNU cohort. (a) The correlation between EMT scores and risk scores for each ACRG subtypes. (b) The correlation between MSI scores and risk scores for each ACRG subtypes.

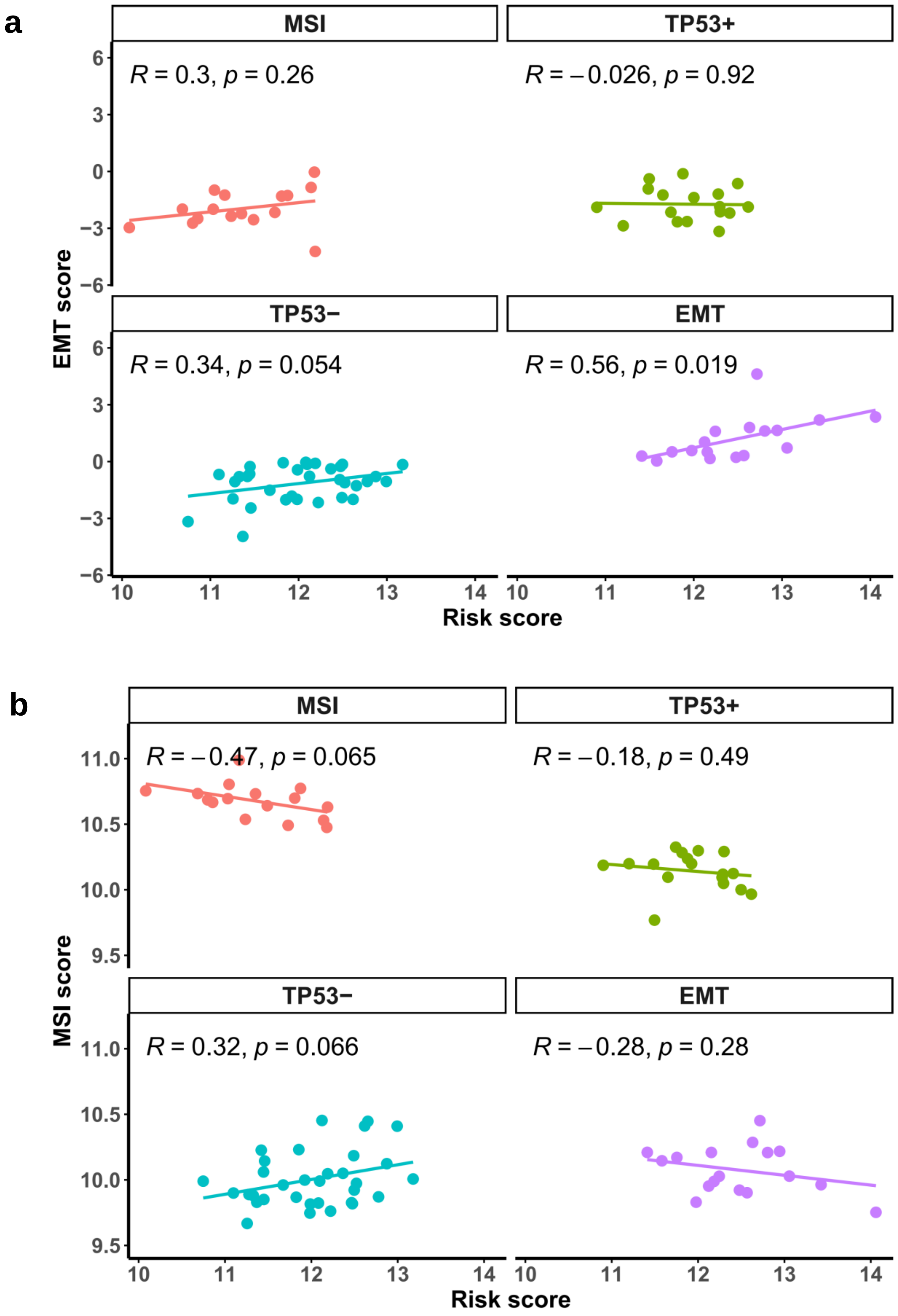

Supplementary Figure S11. Alternative splicing (AS) subtypes with risk score in the SNU cohort. (a) Boxplot of risk scores between AS subtypes. (b) With mean and standard deviation (SD) of risk scores, group was classified as high (>mean+SD), intermediate (between mean+SD and mean–SD), and low risk group (<mean–SD). *P* value presented next to the gene name was calculated between high- and low-risk group with Wilcoxon signed-rank test. EpiS: epithelial subtype, HybS: hybrid subtype, and MesS: mesenchymal subtype.

a

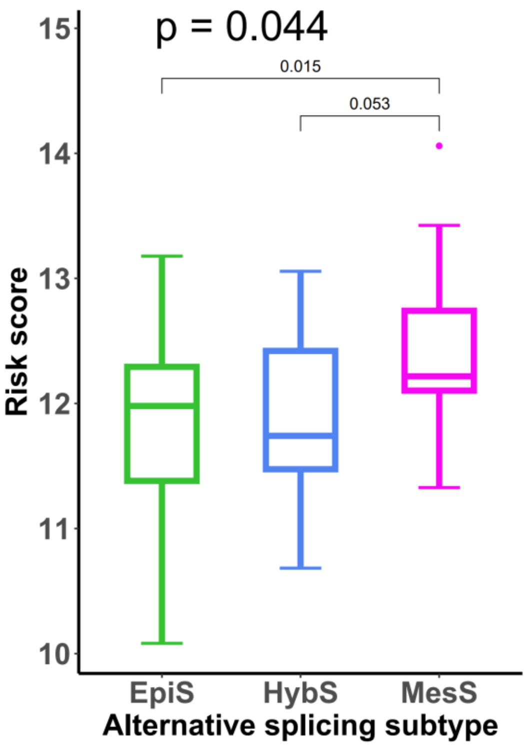

b

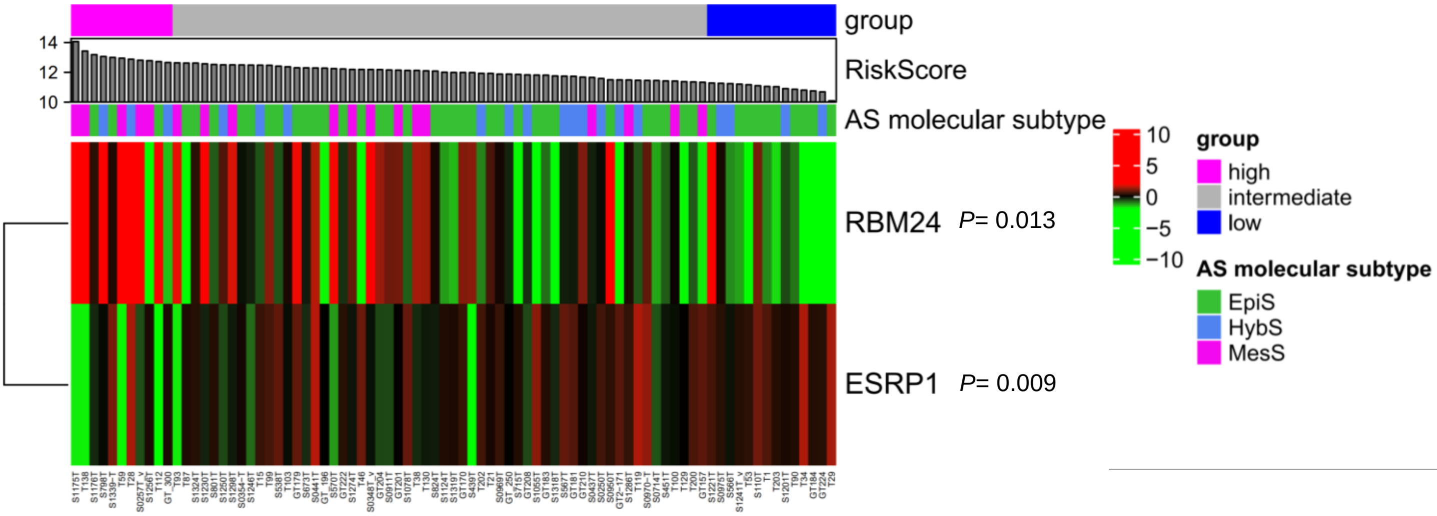

Supplementary Figure S12. Pairwise comparisons between 6-gene based risk group and other molecular subtypes. (a) SNU cohort – TCGA, ACRG, AS, and CGSs. (b) TCGA cohort – TCGA and CGSs. (c) ACRG cohort – ACRG and CGSs.

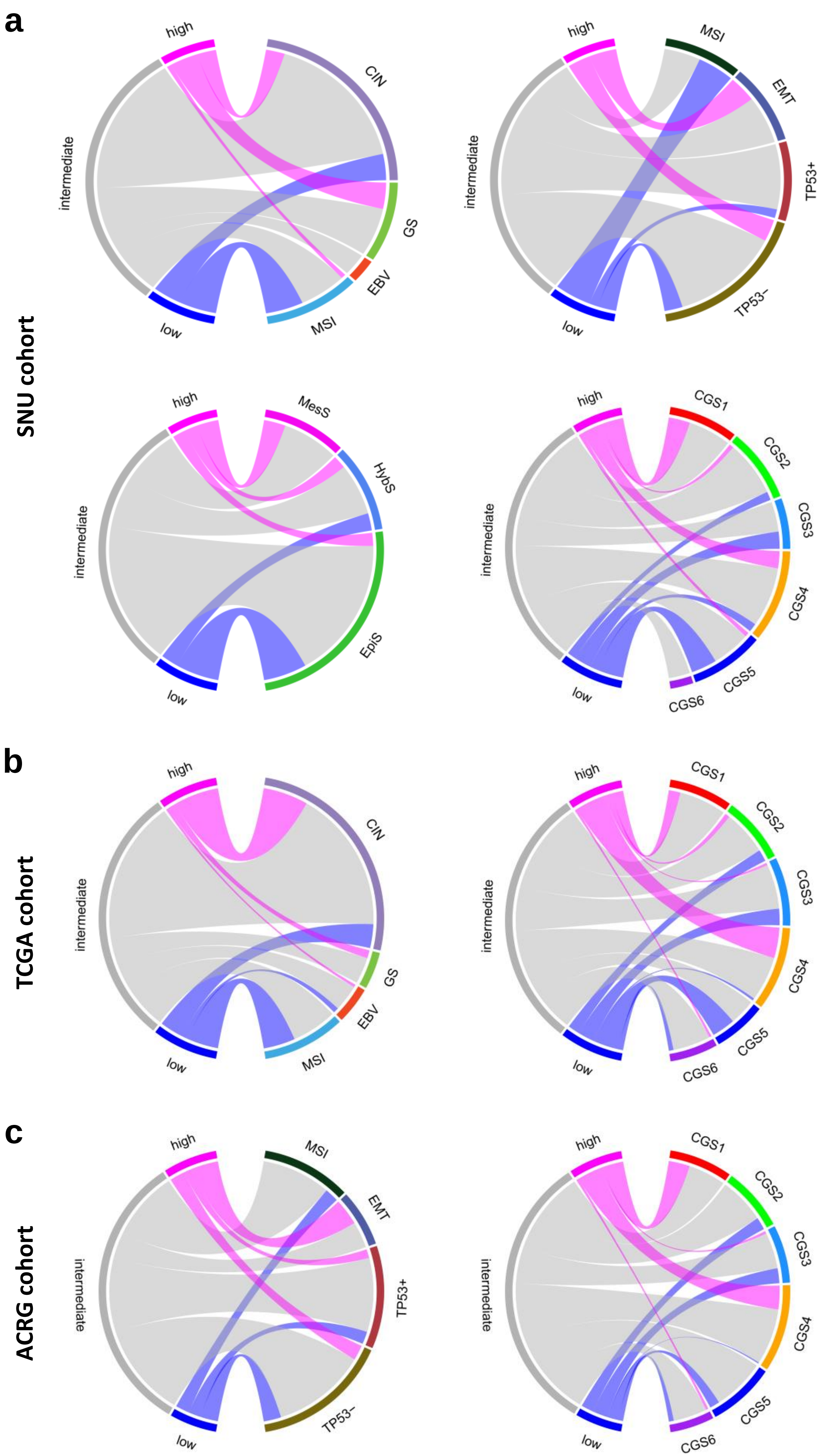

Supplementary Figure S13. Mutation profiles within high- and low-risk groups in SNU and TCGA cohort. Genes that were mutated by more than 40% in each group and six genes (blue rectangle) were presented. If *P*<0.05, it was colored red. (a) SNU cohort. (b) TCGA cohort.

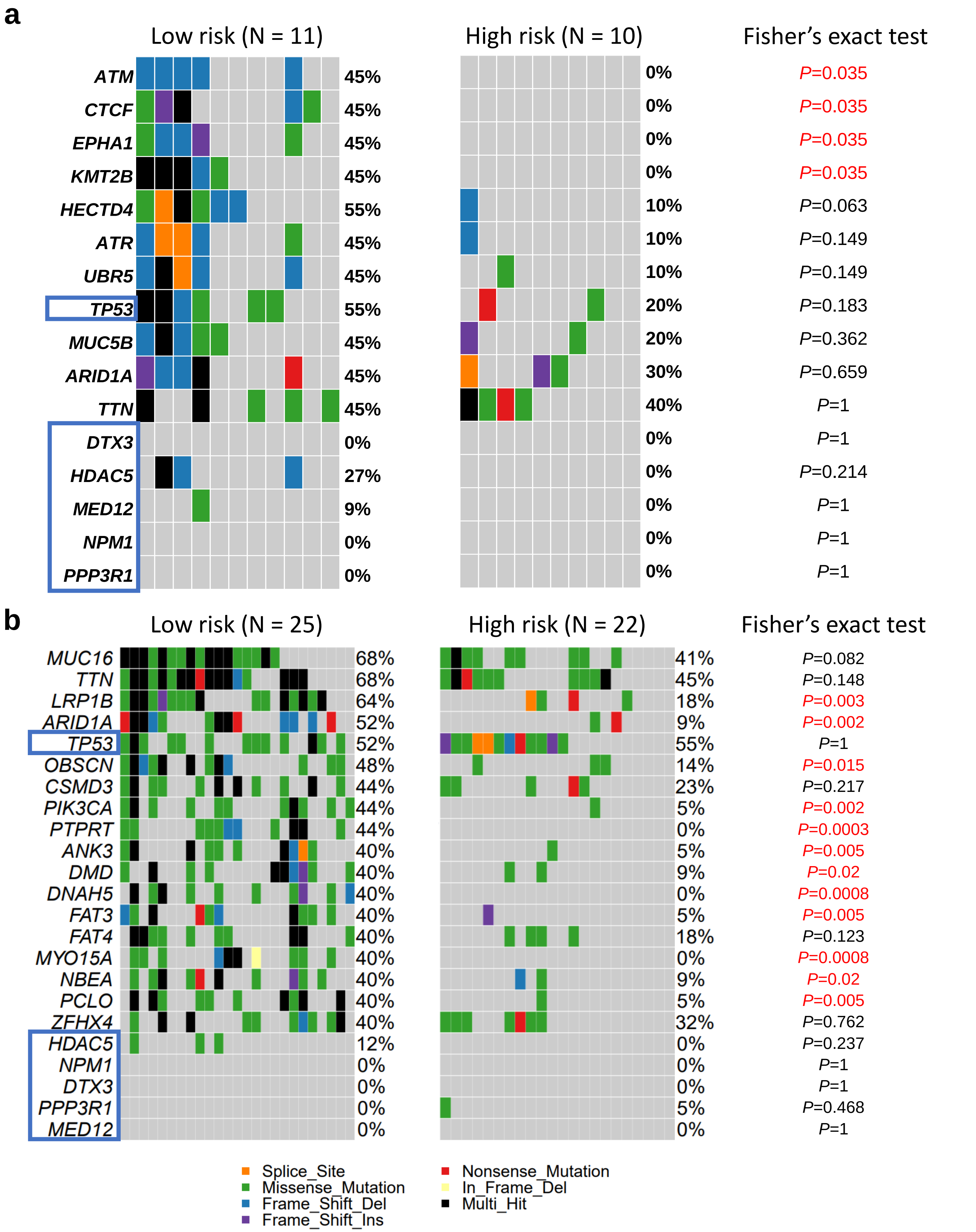

Supplementary Figure S14. Classification of ACRG subtypes in the SNU cohort. (a) Distribution tails of MSI signature and EMT signature in the SNU cohort. (b) TP53 signature status of non-EMT and non-MSI subtype in the SNU cohort (sensitivity = 0.84, specificity = 0.65, and ROC AUC = 0.81).

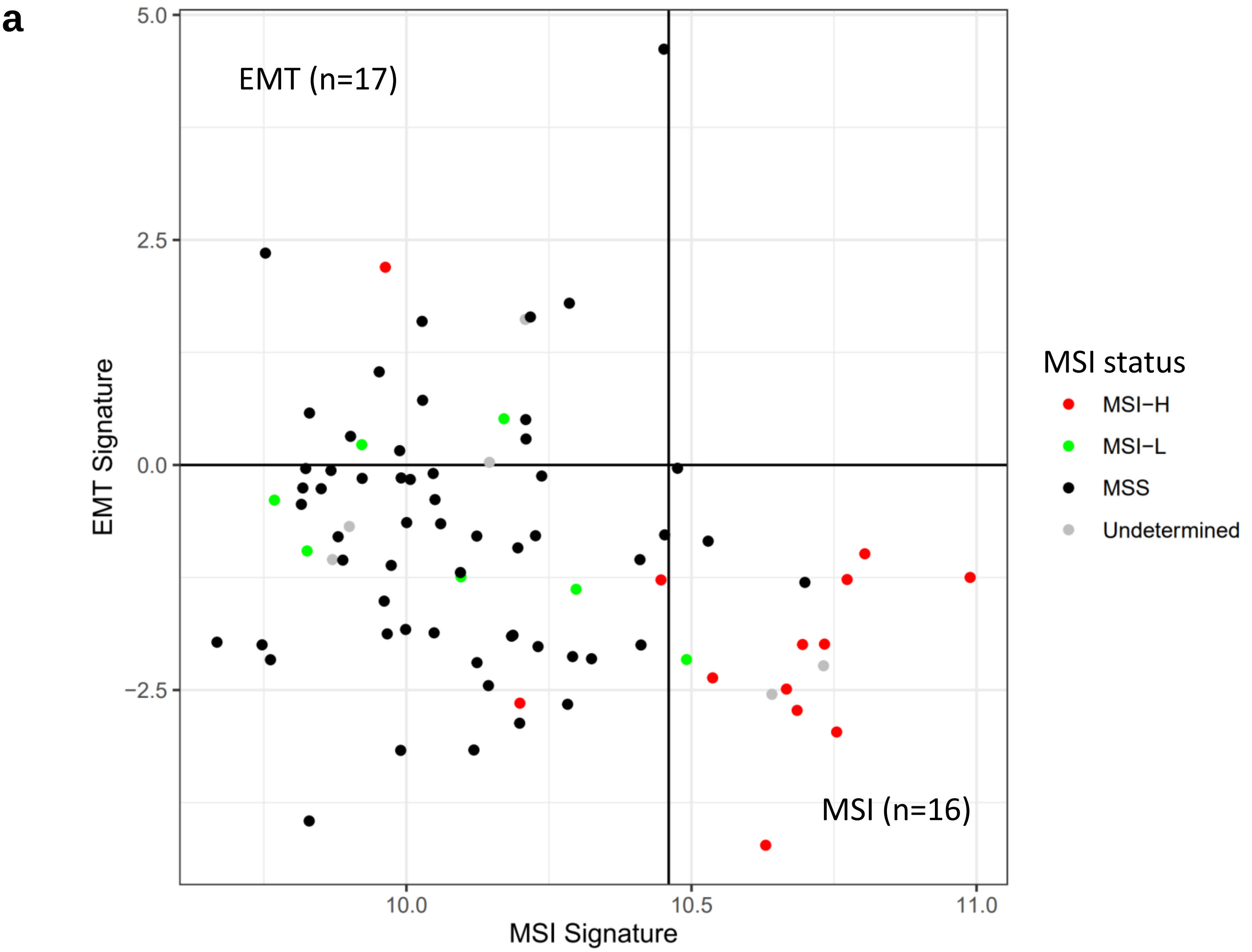

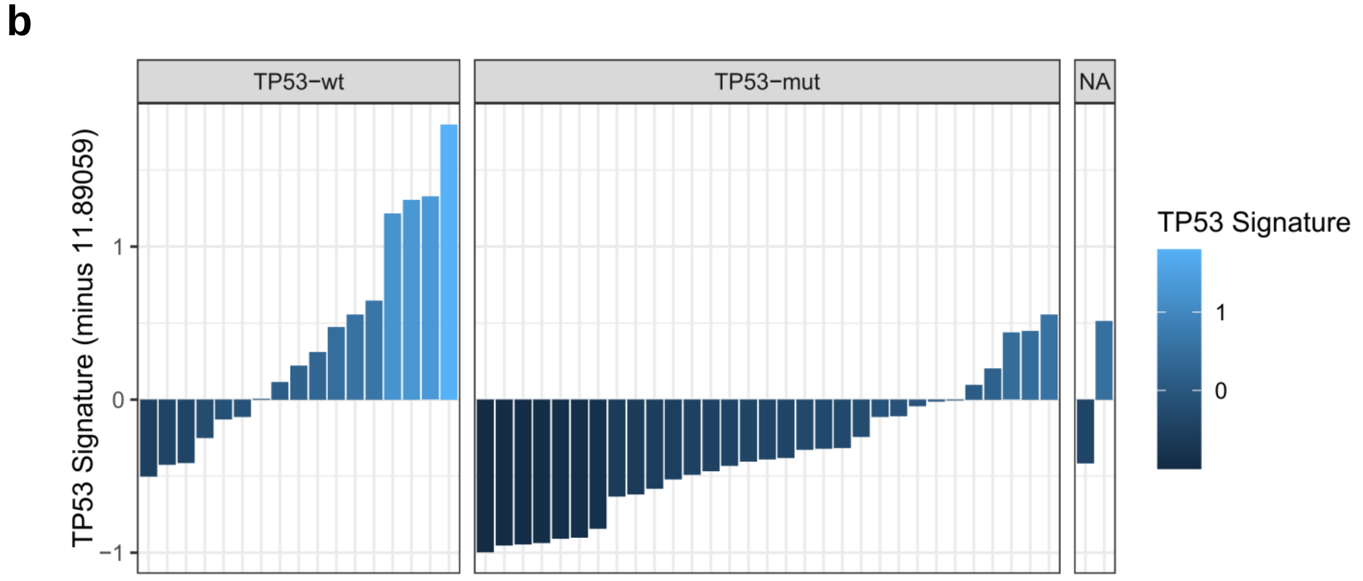

Supplement: Supplementary file 1 — Supplementary Figures [file 41416_2024_2642_MOESM1_ESM.pdf]
