# Supplementary material for: RNA expression of 6 genes from metastatic mucosal gastric cancer serves as the global prognostic marker for gastric cancer with functional validation
Source: Br J Cancer. 2024 Mar 11;130(9):1571–84. doi: 10.1038/s41416-024-02642-6 (PMC11059174; doi:10.1038/s41416-024-02642-6)
Supplement: Supplementary file 2 — Supplementary Tables [file 41416_2024_2642_MOESM2_ESM.pdf]

Supplementary Table S1. Clinicopathologic characteristics between Npos and Nneg tumors.

		NposTumor (n=18)	NnegTumor (n=17)	*P* value
Age (years)		51.6 ± 11.5	50.6 ± 13.3	0.82
Sex				1
	Male	10 (55.6%)	9 (52.9%)	
	Female	8 (44.4%)	8 (47.1%)	
Location				1
	Lower	10 (55.6%)	11 (64.7%)	
	-Middle	5 (27.8%)	4 (23.5%)	
	Upper	3 (16.7%)	2 (11.8%)	
Tumor size (cm)		3.1 ± 1.6	3.0 ± 1.9	0.858
Lauren				0.471
	Diffuse	13 (72.2%)	11 (64.7%)	
	Intestinal	5 (27.8%)	4 (23.5%)	
	Mixed	0 (0.0%)	2 (11.8%)	
WHO differentiation				1
	Moderately differentiated	2 (11.1%)	2 (11.8%)	
	Poorly differentiated	9 (50.0%)	8 (47.1%)	
	Signet ring cell	5 (27.8%)	5 (29.4%)	
	others	2 (11.1%)	2 (11.8%)	
T stage				
	T1a	18 (100.0%)	17 (100.0%)	
N stage				0
	N0	0 (0.0%)	17 (100.0%)	
	N1	13 (72.2%)	0 (0.0%)	
	N2	2 (11.1%)	0 (0.0%)	
	N3	3 (16.7%)	0 (0.0%)	
The number of metastatic lymph nodes		4.2 ± 4.8	0	0.002
The number of retrieved lymph nodes		41.6 ± 9.7	34.1 ± 12.2	0.05

Supplementary Table S2. Canonical Pathway by Ingenuity Pathway analysis with *P* value (adjusted by Benjamini and Hochberg method) < 0.001

Ingenuity Canonical Pathways	-log(B-H p-value)	Ratio	z-score	Molecules
Regulation of the Epithelial-Mesenchymal Transition Pathway	1.15E+01	7.29E-02	NaN	FGF2,FGF7,FZD7,FZD8,HGF,JAG1,MAPK1,PDGFRB,RAF1,RHOA,SMAD2,SMO,WNT4,ZEB2
Phospholipase C Signaling	1.14E+01	6.10E-02	1.069	EP300,GNAQ,GNG7,HDAC10,HDAC11,HDAC5,HDAC6,LAT,MAPK1,PLA2G2A,PPP3R1,RAC3,RAF1,RHOA,SHC1
Cyclins and Cell Cycle Regulation	1.05E+01	1.25E-01	NaN	ABL1,CCND1,CUL1,HDAC10,HDAC11,HDAC5,HDAC6,RAF1,TFDP1,TP53
Axonal Guidance Signaling	9.96E+00	3.63E-02	NaN	ABL1,EFNA3,EFNA5,FZD7,FZD8,GNAQ,GNG7,MAPK1,MMP3,PPP3R1,PRKAR1B,RAC3,RAF1,RHOA,SHC1,SMO,WNT4
Cell Cycle: G1/S Checkpoint Regulation	9.84E+00	1.36E-01	-1.134	ABL1,CCND1,CUL1,HDAC10,HDAC11,HDAC5,HDAC6,TFDP1,TP53
Wnt/β-catenin Signaling	8.80E+00	6.47E-02	1.265	CCND1,CD44,EP300,FZD7,FZD8,GNAQ,SFRP2,SMO,TP53,UBB,WNT4
Adipogenesis pathway	8.64E+00	7.58E-02	NaN	EZH2,FGF2,FZD7,FZD8,HDAC10,HDAC11,HDAC5,HDAC6,SMO,TP53
Ephrin Receptor Signaling	8.64E+00	6.18E-02	0.707	ABL1,ANGPT1,EFNA3,EFNA5,GNAQ,GNG7,MAPK1,RAC3,RAF1,RHOA,SHC1
Telomerase Signaling	8.24E+00	8.57E-02	0.447	ABL1,HDAC10,HDAC11,HDAC5,HDAC6,MAPK1,RAF1,SHC1,TP53
PTEN Signaling	7.57E+00	7.14E-02	0	CBL,CCND1,FOXO4,IGF1R,MAPK1,PDGFRB,RAC3,RAF1,SHC1
Role of NANOG in Mammalian Embryonic Stem Cell Pluripotency	6.54E+00	6.78E-02	0	FZD7,FZD8,MAPK1,RAF1,SHC1,SMO,TP53,WNT4
Endocannabinoid Cancer Inhibition Pathway	5.92E+00	5.59E-02	0.707	CASP10,CCND1,GNAQ,MAPK1,NOS3,PRKAR1B,RAF1,RHOA
Endocannabinoid Developing Neuron Pathway	5.40E+00	6.03E-02	0	CCND1,GNG7,MAPK1,PRKAR1B,RAC3,RAF1,RHOA
Ephrin B Signaling	5.39E+00	8.33E-02	1	CBL,GNAQ,GNG7,MAPK1,RAC3,RHOA
PPARŒ±/RXRŒ± Activation	5.29E+00	4.49E-02	0	EP300,GNAQ,MAPK1,MED12,PRKAR1B,RAF1,SHC1,SMAD2
Oncostatin M Signaling	5.16E+00	1.16E-01	-1.342	MAPK1,MMP3,PLAU,RAF1,SHC1
PI3K/AKT Signaling	5.11E+00	5.38E-02	-1.134	CCND1,MAP3K8,MAPK1,NOS3,RAF1,SHC1,TP53
RAR Activation	5.09E+00	4.19E-02	NaN	EP300,IGFBP3,IL3RA,MAPK1,NSD1,PRKAR1B,RXRG,SMAD2
Human Embryonic Stem Cell Pluripotency	5.07E+00	5.26E-02	NaN	FGF2,FZD7,FZD8,PDGFRB,SMAD2,SMO,WNT4
Androgen Signaling	5.05E+00	5.19E-02	-0.447	CCND1,EP300,GNAQ,GNG7,MAPK1,PRKAR1B,SHC1
STAT3 Pathway	5.05E+00	5.19E-02	-1.633	FGF2,HGF,IGF1R,IL3RA,MAPK1,PDGFRB,RAF1
Calcium Signaling	5.02E+00	4.04E-02	-1	EP300,HDAC10,HDAC11,HDAC5,HDAC6,MAPK1,PPP3R1,PRKAR1B
Cell Cycle: G2/M DNA Damage Checkpoint Regulation	4.95E+00	1.02E-01	0	ABL1,CUL1,EP300,PRKDC,TP53
Actin Cytoskeleton Signaling	4.82E+00	3.74E-02	0	FGF2,FGF7,FN1,MAPK1,RAC3,RAF1,RHOA,SHC1
GŒ±q Signaling	4.75E+00	4.58E-02	0.816	GNAQ,GNG7,MAPK1,PPP3R1,RAC3,RAF1,RHOA
Protein Kinase A Signaling	4.72E+00	2.61E-02	0	DUSP6,GNAQ,GNG7,MAPK1,NOS3,PPP3R1,PRKAR1B,RAF1,RHOA,SMO
Mouse Embryonic Stem Cell Pluripotency	4.64E+00	5.83E-02	0.816	FZD7,FZD8,MAPK1,RAF1,SMO,TP53
IGF-1 Signaling	4.60E+00	5.71E-02	-0.447	IGF1R,IGFBP3,MAPK1,PRKAR1B,RAF1,SHC1
PCP pathway	4.59E+00	8.33E-02	2.236	FZD7,FZD8,RHOA,SMO,WNT4
Opioid Signaling Pathway	4.45E+00	3.28E-02	-0.707	EP300,GNG7,MAPK1,NOS3,PPP3R1,PRKAR1B,RAC3,RAF1
NGF Signaling	4.42E+00	5.26E-02	-0.816	MAP3K8,MAPK1,RAF1,RHOA,SHC1,TP53
Sphingosine-1-phosphate Signaling	4.40E+00	5.22E-02	-0.447	CASP10,GNAQ,MAPK1,PDGFRB,RAC3,RHOA
Glucocorticoid Receptor Signaling	4.38E+00	2.69E-02	NaN	EP300,HSPA2,MAPK1,PBX1,PLAU,PPP3R1,RAF1,SHC1,SMAD2
Cholecystokinin/Gastrin-mediated Signaling	4.38E+00	5.13E-02	0.447	GNAQ,MAPK1,RAC3,RAF1,RHOA,SHC1
G Beta Gamma Signaling	4.29E+00	4.92E-02	0	GNAQ,GNG7,MAPK1,PRKAR1B,RAF1,SHC1
Synaptic Long Term Potentiation	4.23E+00	4.76E-02	-0.816	EP300,GNAQ,MAPK1,PPP3R1,PRKAR1B,RAF1
ERK/MAPK Signaling	4.23E+00	3.63E-02	-0.378	DUSP6,MAPK1,PLA2G2A,PRKAR1B,RAC3,RAF1,SHC1
Notch Signaling	4.17E+00	1.08E-01	NaN	DTX3,JAG1,MAML2,NUMBL
CREB Signaling in Neurons	4.10E+00	3.40E-02	-1.342	EP300,GNAQ,GNG7,MAPK1,PRKAR1B,RAF1,SHC1
Estrogen Receptor Signaling	4.10E+00	4.38E-02	NaN	EP300,MAPK1,MED12,PRKDC,RAF1,SHC1
Insulin Receptor Signaling	4.10E+00	4.41E-02	0	CBL,FOXO4,MAPK1,PRKAR1B,RAF1,SHC1
Integrin Signaling	4.10E+00	3.40E-02	-0.378	ABL1,ITGA8,MAPK1,RAC3,RAF1,RHOA,SHC1
Corticotropin Releasing Hormone Signaling	4.04E+00	4.26E-02	-0.816	GNAQ,MAPK1,NOS3,PRKAR1B,RAF1,SMO
VEGF Family Ligand-Receptor Interactions	4.02E+00	5.88E-02	-1.342	MAPK1,NOS3,PLA2G2A,RAF1,SHC1
FGF Signaling	4.02E+00	5.88E-02	-2.236	FGF2,FGF7,HGF,MAPK1,RAF1
PDGF Signaling	3.99E+00	5.75E-02	-1.342	ABL1,MAPK1,PDGFRB,RAF1,SHC1
Œ±-Adrenergic Signaling	3.86E+00	5.38E-02	NaN	GNAQ,GNG7,MAPK1,PRKAR1B,RAF1
Melanocyte Development and Pigmentation Signaling	3.82E+00	5.26E-02	-1.342	EP300,MAPK1,PRKAR1B,RAF1,SHC1
p53 Signaling	3.76E+00	5.10E-02	NaN	CCND1,EP300,PCNA,PRKDC,TP53
PPAR Signaling	3.72E+00	4.95E-02	0.447	EP300,MAPK1,PDGFRB,RAF1,SHC1
Sumoylation Pathway	3.72E+00	4.95E-02	0	EP300,PCNA,RAC3,RHOA,TP53
GADD45 Signaling	3.68E+00	1.58E-01	NaN	CCND1,PCNA,TP53
GNRH Signaling	3.66E+00	3.53E-02	-0.816	GNAQ,GNG7,MAP3K8,MAPK1,PRKAR1B,RAF1
RhoGDI Signaling	3.58E+00	3.39E-02	NaN	CD44,EP300,GNAQ,GNG7,RAC3,RHOA
HGF Signaling	3.55E+00	4.50E-02	-2.236	CCND1,HGF,MAP3K8,MAPK1,RAF1
Synaptic Long Term Depression	3.55E+00	3.31E-02	-0.816	GNAQ,IGF1R,MAPK1,NOS3,PLA2G2A,RAF1
G-Protein Coupled Receptor Signaling	3.49E+00	2.59E-02	NaN	DUSP6,GNAQ,MAP3K8,MAPK1,PRKAR1B,RAF1,SHC1
ErbB2-ErbB3 Signaling	3.39E+00	6.15E-02	-1	CCND1,MAPK1,RAF1,SHC1
GŒ±i Signaling	3.36E+00	4.00E-02	0.447	GNG7,MAPK1,PRKAR1B,RAF1,SHC1
Endocannabinoid Neuronal Synapse Pathway	3.34E+00	3.94E-02	1.342	GNAQ,GNG7,MAPK1,PPP3R1,PRKAR1B
Melatonin Signaling	3.29E+00	5.71E-02	NaN	GNAQ,MAPK1,PRKAR1B,RAF1
AMPK Signaling	3.24E+00	2.83E-02	-1.342	CCND1,EP300,FOXO4,MAPK1,NOS3,PRKAR1B
Synaptogenesis Signaling Pathway	3.20E+00	2.27E-02	-0.378	EFNA3,EFNA5,MAPK1,PRKAR1B,RAF1,RHOA,SHC1
G Protein Signaling Mediated by Tubby	3.19E+00	1.00E-01	NaN	ABL1,GNAQ,GNG7
VDR/RXR Activation	3.16E+00	5.19E-02	NaN	EP300,IGFBP3,KLF4,RXRG
Erythropoietin Signaling	3.16E+00	5.19E-02	NaN	CBL,MAPK1,RAF1,SHC1
Chemokine Signaling	3.16E+00	5.19E-02	0	GNAQ,MAPK1,RAF1,RHOA
FLT3 Signaling in Hematopoietic Progenitor Cells	3.10E+00	5.00E-02	0	CBL,MAPK1,RAF1,SHC1
Relaxin Signaling	3.09E+00	3.40E-02	-1	GNAQ,GNG7,MAPK1,NOS3,PRKAR1B
JAK/Stat Signaling	3.09E+00	4.94E-02	0	GNAQ,MAPK1,RAF1,SHC1
Prolactin Signaling	3.07E+00	4.88E-02	NaN	EP300,MAPK1,RAF1,SHC1
PEDF Signaling	3.06E+00	4.82E-02	-2	MAPK1,RAF1,RHOA,TP53

Supplementary Table S3. Leave-one-out cross-validation of prediction models.

Model	Predicted	Reference		Sensitivity	Specificity	Positive Predicted Value	Negative Predicted value	Balanced Accuracy
		Nneg	Npos					
SPLS-DA with 13 genes	Nneg	15	5	0.7222	0.8824	0.8667	0.75	0.8023
	Npos	2	13					
SPLS-DA with 6 classifiers	Nneg	15	4	0.7778	0.8824	0.8750	0.7895	0.8301
	Npos	2	14					
Random Forest with 6 classifiers	Nneg	16	2	0.8889	0.9412	0.9412	0.889	0.915
	Npos	1	16					

*Nneg indicated samples without lymph node metastasis, and Npos those with lymph node metastasis.

Supplementary Table S4. Variable importance of Random Forest model.

Classifiers	Importance
DTX3	34.3330111
HDAC5	63.9607944
MED12	-31.040171*
NPM1	48.94107
PPP3R1	6.13948973
TP53	-15.091141*

*the value was multiplied by -1 to original importance.

Supplementary Table S5. Customized 30 genes for nCounter PanCancer Pathways panel.

SALL4	GFAT1	MTDH
CXCL1	B7H1	TAZ
RASSF8	CXCR4	CXCR1
ZEB2	ZEB1	ROR1
F13B	HK2	LGALS4
ICAM1	LATS1	SOX2
LGR5	CD44	PINX1
GDF15	CXCR7	CIP2A
CREB1	PRC1	PADI2
ING5	NR1H4	PAI1

Supplementary Table S6. The sgRNA sequence for target gene Knockout.

	gene	ID	sequence
1	MED12	MED12-hA_28741	AGGATTGAAGCTGACGTTCT
2	TP53	TP53_hA3_51049	CCCCGGACGATATTGAACAA
3		NTC_001*	ACGGAGGCTAAGCGTCGCAA

* Non Targeting Control Guide For Human_0001(HGLibA_64384)

Supplementary Table S7. List of primers used for RT-qPCR

gene	Primer	sequence	Reference
CDH1	CDH1_F	GAACAGCACGTACACAGC CCT	PMID: 12414534
	CDH1_R	GCAGAACTGTCCCTGTCCCAG	
CDH2	CDH2_F	CCACCTTAAAATCTGCAGGC	PMID: 23178117
	CDH2_R	GTGCATGAAGGA CAGCCTCT	
VIM	VIM_F	CTTCAGAGAGAG GAAGCCGA	PMID: 23178117
	VIM_R	ATTCCACTT TGCGTTCAAGG	
Snail1	SNAI1_F	TGCAGGACTCTAATCCAAGTTTAC	PMID: 12414534
	SNAI1_F	GTGGGATGGCTGCCAGC	
Zeb1	Zeb1_F	GCACCTGAAGAGGACCAGAG	PMID: 30862883
	Zeb1_R	TGCATCTGGTGTTCCATTTT	
GAPDH	GAPDH-F	TGCACCACCAACTGCTTA	PMID: 20507635
	GAPDH-R	GGATGCAGGGATGATGTTC	

Supplementary Table S8. Follow up period for survival analysis in SNU, TCGA and ACRG cohorts.

	Overall survival (months)		Recurrence or progression free survival (months)	
	Median	Range	Median	Range
SNU	22.1	3.3-108.6	19.7	1.2-108.6
TCGA	19.6	2.1-122.3	14.5	0-122.3
ACRG	58.9	2.7-105.7	26.6	0.4-100.9
